# On-Limb Orbiting Robot: Proprioceptive Diameter Estimation and Orthogonal Grip–Orbit Control

**DOI:** 10.3390/biomimetics11090636

**Published:** 2026-09-05

**Authors:** Luz M. Tobar-Subía-Contento, Juan A. Cabrera, Anthony Mandow, Jesús M. Gómez-de-Gabriel

**Affiliations:** 1 Facultad de Ingeniería en Ciencias Aplicadas (FICA), Universidad Técnica del Norte, Ibarra 100105, Ecuador; 2 Mechanical Engineering Department, Institute for Mechatronics Engineering and Cyber-Physical Systems (IMECH.UMA), Universidad de Málaga, 29070 Malaga, Spain; jcabrera@uma.es; 3 Systems Engineering and Automation Department, Institute for Mechatronics Engineering and Cyber-Physical Systems (IMECH.UMA), Universidad de Málaga, 29070 Malaga, Spain; amandow@uma.es

**Keywords:** active haptic perception, enclosure grasping, bio-inspired locomotion, grasping force control, physical human–robot interaction, contact stability, wearable robotics, on-body locomotion

## Abstract

On-body robots that travel around a human limb must keep a firm enough grip to avoid slipping or detaching, while never pressing hard enough to hurt—a balance that is hardest to strike precisely when the robot is orbiting the limb and gravity continually redistributes the contact loads. This paper presents an open, non-anthropomorphic robot that wraps around a compliant cylindrical surface with a three-contact grasp: a central traction module with two in-line driven wheels, and two lateral spring-loaded arms with distal wheels. Its central contribution is an actuation-space decomposition in which the two lateral wheel torques, expressed in a common-mode/differential basis, simultaneously drive the orbital motion and regulate the central normal force. We show that this basis diagonalises both the rolling kinematics and the static force balance, so the differential (grip-regulating) channel is provably orthogonal to the common-mode (propulsion) channel: a single pair of actuators perform both tasks without mutual interference and without a dedicated force mechanism. A model-based feedforward law derived from the static contact model, corrected by a PI term fed back from the compliant arms—which double as the force sensor—keeps the central force within a safe band; in a full-revolution simulation the differential command reverses sign to counteract the gravitational load swing while leaving the orbit undisturbed. The same compliant arms yield a closed-form estimate of the cylinder radius and contact geometry, accurate to below one millimetre across a 45–87 mm diameter range, from proprioception alone. Preliminary prototype tests reproduce the predicted behaviour, supporting the approach for future wearable and assistive applications.

## 1. Introduction

The emergence of on-body robotic systems represents a significant frontier in wearable technology, with potential applications spanning from continuous health monitoring to active physical assistance [1]. Unlike traditional industrial manipulators that operate on rigid workpieces, robots designed for on-limb locomotion must navigate compliant, non-standardised, and dynamic surfaces, such as human skin and soft tissues [2]. This inherent non-rigidity poses a dual challenge: maintaining sufficient contact force for stable traction while ensuring that the pressure exerted remains within safe limits for potential use on human limbs.

This challenge, and our solution to it, are closely mirrored in biology. When a person grips someone else’s forearm without looking—to guide, steady, or reposition them—they estimate its girth and taper directly from the way the hand wraps around it, and they slide the grip along the limb while continuously adjusting the squeeze to the changing thickness. This is active haptic perception: in the classification of exploratory procedures by Lederman and Klatzky [3], the enclosure procedure—wrapping the hand around an object—is precisely the movement humans use to extract an object’s global shape and size without vision, while pressure regulates grip force against slippage. The robot proposed here is a mechanical analogue of enclosure: it wraps a three-contact grip around the limb, infers the local diameter from the grasp configuration alone (no external ranging sensor), and modulates its normal force as it travels. A comparable strategy appears in legless arboreal locomotion: snakes climbing cylinders of widely varying diameter grip by friction, generating just enough muscular normal force to avoid slipping and typically keeping a safety margin above that minimum rather than gripping maximally [4]. Our controller adopts the same principle—a normal force held inside a safe band, neither so low that contact is lost nor so high that the surface is harmed—but achieves it on a wheeled orbiting platform rather than an elongate body.

**Figure 1:**
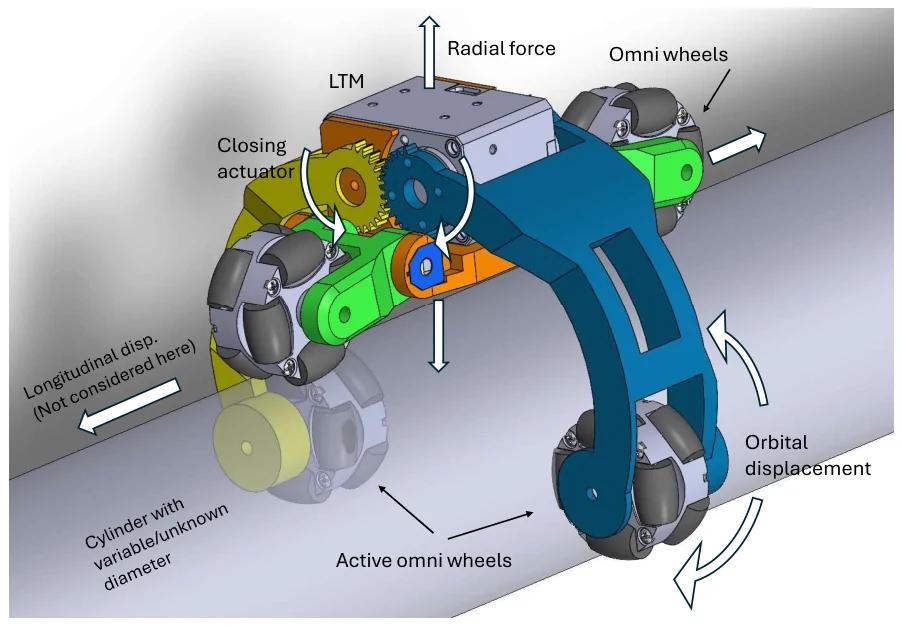
Design of the on-body robotic system, featuring a mobile platform (MP) with two in-line drive wheels mounted on the green arms for locomotion over cylindrical surfaces. The robot includes two lateral arms, shown in yellow and blue, with distal wheels, enabling adaptive grasping and radial force regulation. Redundant actuation through both arm articulation and wheel contact enables contact stability while limiting pressure on human limbs.

Current state-of-the-art solutions for on-body mobility often rely on specialised adhesion mechanisms [5] or closed-structure bracelet designs [6,7], which may limit flexibility or require manual donning. To address these challenges, this study presents a novel, non-anthropomorphic robotic prototype designed for on-limb applications, focusing on adaptive grasping and stable human–robot interaction (see Figure 1). To satisfy the dual requirement of sufficient traction and bounded contact pressure simultaneously, the proposed system exploits the geometry of a three-contact grasp: the lateral wheel torques are decomposed into a common-mode component that compensates gravity and drives circumferential motion, and a differential component that regulates the normal force at the central traction module—both acting simultaneously on the same two actuators without mutual interference.

The significance of this design over existing methods is characterised by:

- **On-body Adaptive Grasping:** The robot is specifically engineered for embodied, human-interactive applications, moving beyond environmental object manipulation.
- **Non-Anthropomorphic Compliance:** The use of a two-link arm for real-time grasping force estimation balances mechanical simplicity with functional adaptability [8].
- **Actuator Reuse for Radial Contact Force Control:** The lateral wheels serve dual roles—orbital propulsion under symmetric actuation, and central contact regulation under asymmetric actuation—reducing mechanical complexity while maintaining active radial force control without a dedicated mechanism.
- **Design for Compliant Surfaces:** The contact and control architecture do not presuppose a rigid substrate, targeting eventual use on soft, non-standardised surfaces such as human skin; the tests reported in this paper, however, use a rigid cylinder (Section 8.4), and validation on compliant, non-rigid bodies remains future work (Section 9).

Among the main contributions of this work include the following:

- **Actuation-Space Decomposition:** The two lateral wheel torques are written in a common-mode/differential basis that *diagonalises* both the velocity map and the static force map. The common-mode command $\tau_{\Sigma }=\tau_{w_{1}}+\tau_{w_{2}}$ compensates gravity and drives the orbital motion; the differential command $\Delta \tau_{d}=\tau_{w_{1}}-\tau_{w_{2}}$ regulates the central normal force $f_{n_{0}}$, computed in feedforward from the static model and corrected by a PI term fed back from the compliant central contact. Because the two channels are orthogonal by construction, they are superimposed on the same two actuators without mutual interference, eliminating the need for a dedicated radial force mechanism.
- **Orbital Stability Bounds:** A closed-form, orientation-dependent lower bound on the grasping torque that guarantees non-detachment and slip-free rolling over the full $360^{\circ }$ orbit under gravity-induced load redistribution.
- **Proprioceptive Geometry Estimation:** A closed-form estimator of the cylinder radius and of the three contact points from two already-available proprioceptive signals (the arm encoder and the compliant swing-link deflections), with a sensitivity analysis showing sub-millimetre accuracy across the whole 45–87 mm diameter range.

The objective of this study is to provide a control framework for grasping force regulation and contact-consistency assessment during interaction with the manipulated surface.

## 2. Related Work

### 2.1. On-Body Mobile Robots

Locomotion on flexible or loose surfaces has been explored through various mechanisms. Cloth-climbing robots like *Clothbot* [9], *Rubbot* [10], and *Rovables* [11] exploit fabric deformability, while systems like *CLASH* [5] utilise gecko-inspired adhesion. While effective for clothing, these are often unsuitable for direct skin contact. Alternatives include track systems mounted on textiles [12,13], though these rely on predefined paths. Bare skin navigation has been addressed by *SkinBot* [1,2] via suction-based designs, though tethering remains a constraint.

A related bio-inspired thread concerns bodies that locomote *around* cylindrical substrates by gripping them. Snake robots reproducing arboreal concertina locomotion wrap and unwrap their body around branches and poles, modulating grip to the substrate diameter [14]; the underlying biological gripping strategy trades economy for a safety margin in normal force [4]. Our platform pursues the same enclose-and-regulate principle with a compact three-contact wheeled grip rather than an elongate body, trading whole-body compliance for mechanical simplicity. The problem of holding a mobile robot against a surface by regulating contact normal forces in real time also arises in legged climbers: a bio-inspired robot gecko, for instance, senses the normal force at each foot and adapts its compliance on the fly to attach and detach without losing contact as it moves [15]. Our robot addresses the analogous problem in a different regime—a continuous orbital roll around a compliant limb rather than a discrete foot gait—and, crucially, obtains its contact force feedback from the same compliant elements that carry the wheels, without a dedicated force sensor at the contact.

In contrast, on-limb mobile robots utilise grasping forces to operate on bare skin or tight clothing. While pneumatic textile robots have been proposed [16], most rely on rigid mechanisms driven by electric motors. Existing designs include closed-bracelet structures like *Movelet* [6] or capsule-based robots [7], both of which require manual donning. The proposed open-structure design builds upon earlier versions [17] that used position control and torsion springs, enabling autonomous attachment and real-time adjustment during navigation. Those earlier versions established diameter-adaptive grasping through arm position control, but did not address propulsion: the platform could grip a limb of varying diameter, but not orbit around it while doing so. The present contribution is specifically the coupling of the two tasks on a single actuator pair, made possible by the common-mode/differential decomposition of Section 4.

The actuation-space decomposition itself is, in structure, a change of basis in the spirit of the classical operational-space formulation [18] and of hybrid position/force control [19], both of which separate a manipulator’s task directions into independently controlled motion and force subspaces. The present setting differs from that literature in two respects that motivate a dedicated derivation rather than a direct application of either framework. First, the two channels are not associated with two different generalised coordinates of a single point (as in hybrid position/force control) but with two physically distinct actuation channels—common-mode and differential wheel torque—acting through a fixed, non-redundant 2 × 2 mapping without the kinematic redundancy that operational-space methods are primarily designed to resolve. Second, the “force” direction being regulated is not the interaction force at the end-effector performing the task, but the normal preload at a third, passive contact (the central traction module) that is mechanically coupled to the two active contacts only through the rigid-body closure constraint of Section 4.5; the decomposition therefore serves contact maintenance at an unactuated point, rather than task-space force control at the actuated one.

### 2.2. Compliance in Physical Human–Robot Interaction (pHRI)

Compliance is a central requirement for robotic systems intended to interact with humans or deformable environments. In manipulation tasks, compliant behaviour contributes to safer contact, reduces the risk of excessive pressure, and improves robustness against geometric uncertainty. These aspects are critical when navigating the human limb, as the interaction must accommodate soft tissues and posture-dependent geometry.

In the context of on-body locomotion, compliance must be understood as an active control problem: the robot must maintain sufficient normal force to preserve traction without causing damage or discomfort. This work distinguishes two regulation tasks—the lateral grasping force set by the arm actuator, and the central normal force set by the differential wheel torque—and, unlike prior designs, achieves the second by *reusing* the locomotion actuators rather than adding a dedicated mechanism.

The way contact force is sensed here also follows an established principle. Series elastic actuation deliberately places a compliant element between actuator and load and infers force from its measured deflection, converting force sensing into an angular measurement [20]; the same idea underpins proprioceptive actuator designs in legged robots, where contact forces are recovered from the transmission itself instead of from a sensor at the foot [21]. The spring-loaded swing links of the central traction module play that role in the present platform, with the difference that the compliant element carries the wheel rather than sitting in the drive train, so the deflection reports the contact normal force directly. The compliant central mechanism doubles as the force sensor that closes this loop, so no separate force transducer is required.

## 3. Robot Design

The proposed robotic system is a non-anthropomorphic mobile platform designed for autonomous navigation on compliant cylindrical structures, such as human limbs or industrial piping. The device architecture prioritises mechanical simplicity and active safety to ensure stable physical human–robot interaction (pHRI).

### 3.1. Mechanical Architecture

The robot’s chassis, referred to as the mobile platform (MP), is the payload and provides structural rigidity with lightweight requirements. The system is composed of two primary functional modules:

- **Central Traction Module:** Located at the centre of the MP, this module consists of two in-line drive wheels equipped with a suspension system. It is responsible for the primary forward and backward locomotion along the cylinder’s longitudinal axis.
- **Lateral Grasping Arms:** Two symmetric arms are pivotally attached to the central body, each featuring a distal omnidirectional wheel at its terminal end. A single high-torque servomotor drives both arms through a gear coupling that constrains them to rotate in opposite directions by equal magnitude, so that a single actuator torque $\tau_{a}$ is inherently split equally between the two proximal joints. This mechanical coupling guarantees grasp symmetry by construction, without requiring separate actuation or synchronisation between the two arms [8].

### 3.2. Actuation and Sensing

The system employs a redundant actuation strategy to decouple locomotion from contact stability:

- **Grasping Actuation:** A high-torque servomotor regulates the opening and closing of the lateral arms providing mostly normal forces. By modulating the proximal joint angle, the robot can adapt to varying limb diameters [17]. The grasping actuation is completed with assistance of the distal wheels, providing tangential forces. The controlled combination of normal and tangential forces makes this grasping system adapt to different grasping requirements.
- **Locomotion Actuation:** The omnidirectional wheels (front, rear, and two sides) enable independent movement. While the central traction module drives longitudinal motion, the lateral wheels allow for circumferential rolling around the limb without compromising the grip.
- **Compliant Sensing:** The two in-line wheels of the central traction module are carried on swing links preloaded by rotational springs. The deflection of each link is measured by an angular sensor ($\theta_{0,f}$ at the front and $\theta_{0,b}$ at the rear), so that the spring characteristic converts their combined deflection directly into an estimate of the central normal force $f_{n_{0}}$ (Section 8.1). The two readings are generally different, but their difference is a longitudinal (pitch) effect: in the transverse plane there is a single contact $P_{c_{0}}$, and therefore a single offset and a single force estimate, both obtained from the mean of the two sensors. This mechanism therefore serves a dual purpose: it acts as the force sensor that closes the radial force loop (Section 7.2) and, through the static model, provides an estimate of the grasping normal force at the lateral contacts; the price is that the central contact point is no longer fixed, since the swing links allow it to move radially, as modelled in Section 4.

### 3.3. Functional Interaction Modes

To manage the complexity of navigating a soft, non-rigid environment like a human limb, the device operates through two coordinated modes:

1. **Lateral Grasping:** The lateral arms exert a controlled normal force to achieve a secure wrap-around grasp. This force is regulated based on joint-angle feedback.
2. **Active Radial Contact Force Control:** The central body maintains vertical contact consistency through front and rear wheels, driven by a model-based feedforward command with an optional force feedback correction to ensure the MP wheels receive sufficient normal force during motion.

## 4. Kinematic and Contact Modelling

The vehicle is modelled as a 1-DOF mechanism where the human limb (or cylindrical substrate) acts as a fixed revolute axis. The state is defined by the orbital angle *ϕ*, representing the platform’s angular position in the transverse plane.

### 4.1. Coordinate Systems and Generalised Coordinates

The wheel configuration of the robot is described by the joint-space vector $q={\left[\theta_{1},\;\theta_{2}\right]}^{⊤}\in R^{2}$, where $\theta_{1}$ and $\theta_{2}$ are the angular positions of the two lateral drive wheels. The associated operational-space coordinates are $x={\left[\phi ,\;\gamma \right]}^{⊤}$: the orbital angle *ϕ* describes the rotation of the entire mobile platform (MP) about the cylinder’s longitudinal axis relative to the gravity vector in the transverse plane, and *γ* is the lateral contact angle of Section 4.4, i.e., the angular position of the lateral contacts on the cylinder surface measured from the central contact. The radial position of the platform is *not* an independent coordinate: it is the dependent output $\rho =R+a(\theta_{0})$ recovered from the contact geometry, as shown in Section 4.5. The arm angle $\theta_{a}$ is not an independent joint coordinate in this framework; it is determined by the stability and slip-prevention conditions of Section 7 and acts as a reference input to the grasping actuator. The swing-link deflections $\theta_{0,f}$ and $\theta_{0,b}$ of the central compliant mechanism are instrumented, non-actuated coordinates: they are not commanded, but they are measured, and they play a dual role. Geometrically, their mean sets the radial position of the central contact point $P_{c_{0}}$, which is therefore *not* fixed. Statically, the same mean is mapped by the spring characteristic onto the single central normal force $f_{n_{0}}$ of the transverse plane (Section 8.1), which closes the radial force loop of Section 7.2. Accepting the extra kinematic variable is thus what buys a direct force measurement without adding a dedicated sensor.

### 4.2. Contact Geometry

**Figure 2:**
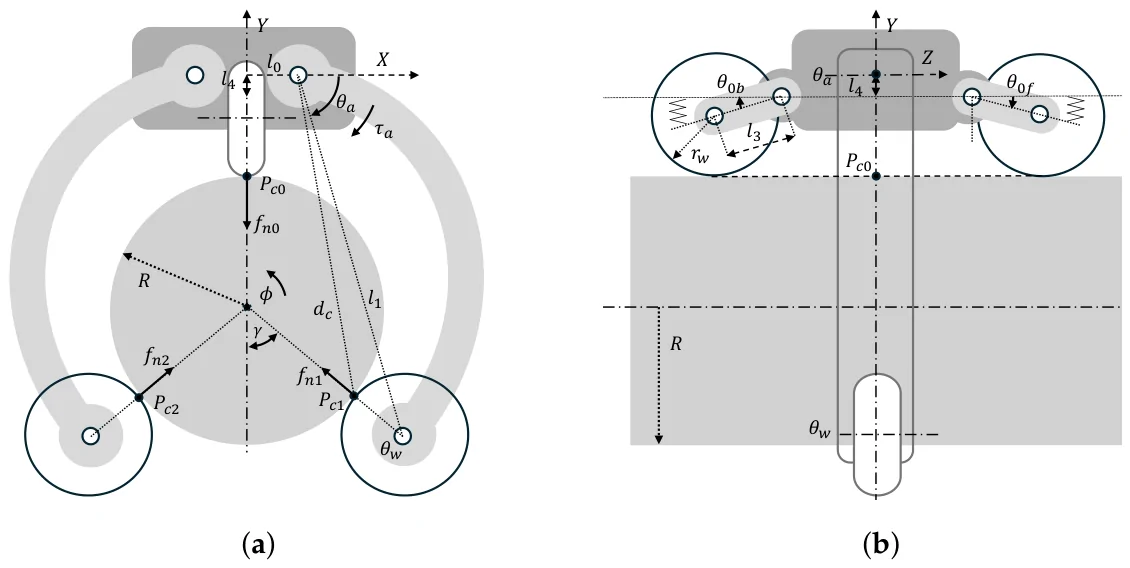
Contact force model of the robot. (**a**) Transversal view, showing the lateral contact angle *γ* at the cylinder centre, the spring-loaded swing links (deflections $\theta_{0,f}$, $\theta_{0,b}$, entering the transverse model only through the averaged contact depth *a*) that carry the central wheel, the central normal force $f_{n_{0}}$, and the lateral tangential forces $f_{t_{1}}$, $f_{t_{2}}$ produced by the wheel torques $\tau_{w_{1}}$, $\tau_{w_{2}}$. (**b**) Longitudinal view.

The robot establishes three primary contact points in the transverse (XY) plane: a central contact $P_{c_{0}}$ associated with the central traction module, and two symmetric lateral contacts $P_{c_{1}}$ and $P_{c_{2}}$, as shown in Figure 2a).

The two in-line wheels of the central traction module are each carried by a spring-loaded swing link, whose deflections $\theta_{0,f}$ (front) and $\theta_{0,b}$ (rear) are measured independently and need not coincide. Their difference is a longitudinal (pitch) effect that lies outside the transverse plane and is not relevant here; what the transverse model requires is the *mean* radial offset introduced by the two links. Collecting both measurements in $\theta_{0}=\left(\theta_{0,f},\theta_{0,b}\right)$, the central module is lumped into a single equivalent wheel, and the central contact point is

(1) $$P_{c_{0}}=\left(0,\;-a(\theta_{0})\right),\;a\left(\theta_{0}\right)=l_{4}+\frac{l_{3}}{2}\left(\sin\theta_{0,f}+\sin\theta_{0,b}\right)+r_{w},$$

where $l_{3}$ is the swing-link length, $l_{4}$ the *y*-offset of its pivot, and the averaging of the two sines is the approximate lumped model of the central contact offset: it collapses the two longitudinal contacts onto the single transverse contact $P_{c_{0}}$ of Figure 2. The *contact depth* $a(\theta_{0})$ is thus a measured quantity, not a constant. The right lateral wheel centre follows from the arm angle $\theta_{a}$ (positive downward), $P_{1}=\left(l_{0}+l_{1}\cos\theta_{a},\;-l_{1}\sin\theta_{a}\right)$, where $l_{0}$ is the arm pivot x-offset and $l_{1}$ is the arm length. By symmetry, $P_{2}=\left(-P_{1x},\;P_{1y}\right)$.

The lateral wheel–cylinder contact points are obtained by offsetting each wheel centre by $r_{w}$ toward the estimated cylinder centre $C=(0,C_{y})$: $P_{c_{1}}=P_{1}+r_{w}n_{1}$ for the right lateral contact, where $n_{1}=\left(C-P_{1}\right)/∥C-P_{1}∥$ is the inward unit normal. By symmetry, $P_{c_{2}}=\left(-P_{c_{1x}},\;P_{c_{1y}}\right)$.

### 4.3. Cylinder Radius Estimation

With the central contact point given by (1), the cylinder centre $C={\left(0,C_{y}\right)}^{⊤}$ satisfies two conditions simultaneously: the central contact lies on the cylinder surface, $|C_{y}+a\left(\theta_{0}\right)|=R$, which gives $C_{y}=-a\left(\theta_{0}\right)-R$, and the lateral wheel is externally tangent to the cylinder, $∥C-P_{1}∥=R+r_{w}$. Substituting $C_{y}=-a\left(\theta_{0}\right)-R$ into the tangency condition and expanding,

(2) $$P_{1x}^{2}+{\left(P_{1y}+a\left(\theta_{0}\right)+R\right)}^{2}={\left(R+r_{w}\right)}^{2},$$

solving for *R* yields the closed-form estimator

(3) $$R_{\mathrm{est}}\left(\theta_{a},\theta_{0}\right)=\frac{r_{w}^{2}-P_{1x}^{2}-{\left(P_{1y}+a\left(\theta_{0}\right)\right)}^{2}}{2\;\left(P_{1y}+a\left(\theta_{0}\right)-r_{w}\right)},$$

with $P_{1x}$ and $P_{1y}$ functions of $\theta_{a}$ alone. The radius therefore depends only on directly measured angles—the arm encoder $\theta_{a}$ and the two swing-link sensors, which enter solely through the averaged contact depth $a(\theta_{0})$—and requires no integration, filtering, or additional hardware, since all three signals are already needed for grasping actuation and force estimation respectively. Once $R_{\mathrm{est}}$ is known, the cylinder centre is $C_{y}=-a\left(\theta_{0}\right)-R_{\mathrm{est}}$ and the lateral contact points $P_{c_{1}}$ and $P_{c_{2}}$ are recovered by projecting $P_{1}$ and $P_{2}$ toward *C* by $r_{w}$.

In practice, $\theta_{a}$ is obtained by closing the lateral arms with torque $\tau_{a}$ until the robot stops moving—the cylinder acts as a mechanical stop that uniquely determines the grasp configuration from the three simultaneous contact constraints. The arm angle at that equilibrium is read from the servo encoder and the swing-link deflections from their angular sensors, and all are substituted into (3). The sensitivity of the estimator to each of the two measurements, and the resulting safe operating range, are analysed in Section 8.2.

### 4.4. Lateral Contact Angle

The same geometry used to estimate *R* also determines the angular position of each lateral contact relative to the central contact, a quantity used to characterise the grasp configuration in Section 8.2. Let *γ* denote the *lateral contact angle*: the angle, measured at the cylinder centre *C*, between the vertical axis (the direction from *C* to the central contact $P_{c_{0}}$) and the line from *C* to a lateral contact point $P_{c_{1}}$ (or, by symmetry, $P_{c_{2}}$), as shown in Figure 2a.

Because $P_{c_{1}}$ lies on the cylinder surface at distance *R* from *C*, along the same direction as the wheel centre $P_{1}$ (offset from $P_{c_{1}}$ by $r_{w}$ toward *C*), the horizontal component of the tangency relation $∥C-P_{1}∥=R+r_{w}$ directly gives

(4) $$\gamma =\arcsin\;\left(\frac{P_{1x}}{R+r_{w}}\right)=\arcsin\;\left(\frac{l_{0}+l_{1}\cos\theta_{a}}{R_{\mathrm{est}}\left(\theta_{a},\theta_{0}\right)+r_{w}}\right),$$

using the radius estimate $R_{\mathrm{est}}\left(\theta_{a},\theta_{0}\right)$ from (3).

*Why γ matters.* Beyond describing the grasp, *γ* orients the contact frame at each lateral wheel and therefore sets how the two available actuation inputs act on the cylinder. At the right lateral contact the inward normal and the tangent are

(5) $$n_{1}=\left(-\sin\gamma ,\;\cos\gamma \right),\;t_{1}=\left(\cos\gamma ,\;\sin\gamma \right),$$

and $n_{2}$ and $t_{2}$ follow by mirror symmetry. The two inputs act along these two orthogonal directions and are physically distinct in origin:

- The **normal force** $f_{n_{i}}$, along $n_{i}$, is produced by the *closing torque of the arms* $\tau_{a}$: the grasping actuator presses each lateral wheel against the surface, and this is the force that cages the cylinder. Its retaining (vertical) component is $f_{n_{i}}\cos\gamma$, so the grasp holds the vehicle only as long as *γ* stays away from $90^{\circ }$.
- The **tangential force** $f_{t_{i}}$, along $t_{i}$ and hence *perpendicular* to $n_{i}$, is produced by the *drive torque of that wheel* $\tau_{w_{i}}$, and is bounded by Coulomb friction, $|f_{t_{i}}|\leq \mu f_{n_{i}}$.

Projecting (5) onto the central contact normal $n_{0}$ yields the two coupling coefficients that govern the whole static model:

(6) $$n_{i}\cdot n_{0}=-\cos\gamma ,\;t_{i}\cdot n_{0}=\mp \sin\gamma .$$

Thus *γ* arbitrates a trade-off between the two actuation mechanisms: the closing torque of the arms retains the robot and loads the central contact in proportion to cosγ, while the wheel torques can modulate that same contact force in proportion to sinγ. Both channels are usable only in the interior of the admissible interval, which motivates the bounds established next. Note that *γ* is fixed once the arms have closed on a given cylinder, so both coefficients are constants of the grasp rather than functions of the orbital angle *ϕ*; this is used throughout Section 6 and Section 7.

*Physical bounds. γ* must remain strictly within $\left(0^{\circ },90^{\circ }\right)$. The lower limit $\gamma \to 0^{\circ }$ corresponds to the lateral contact collapsing onto the vertical axis, where the two lateral wheels would occupy the same position as the central wheel; this degenerate configuration is precluded by the finite wheel width and the minimum-diameter limit $\theta_{a,\max}$ already established in Section 8.2. The upper limit $\gamma =90^{\circ }$ corresponds to the lateral contact reaching the cylinder equator ($D=D_{\max}$): beyond this angle the grasp can no longer support the vehicle’s weight against gravity during orbital rotation, and the robot risks tipping or falling off the cylinder. For the valid range $\theta_{a}\in \left[55.5^{\circ },84.4^{\circ }\right]$ established in Section 8.2, *γ* spans approximately $\left[27^{\circ },90^{\circ }\right)$ for this prototype, confirming that the grasp remains within the safe angular range across all admissible cylinder diameters.

### 4.5. Operational-Space Velocity Mapping and Force Duality

The lateral wheels roll directly on the cylinder surface, so their rolling radius is the cylinder radius *R* itself. Crucially, the two lateral contacts are *not* pinned to the surface: by the definition of *γ*, contact $P_{c_{1}}$ sits at arc angle ϕ+γ from the central contact and $P_{c_{2}}$ at ϕ−γ. Rolling without slipping therefore constrains each wheel to the arc travelled by its own contact,

(7) $$r_{w}\;{\dot{\theta }}_{1}=R\;\left(\dot{\phi }+\dot{\gamma }\right),\;r_{w}\;{\dot{\theta }}_{2}=R\;\left(\dot{\phi }-\dot{\gamma }\right),$$

which inverts into a square operational Jacobian $J\in R^{2\times 2}$,

(8) $$\left[\begin{array}{c} \dot{\phi }\\ \dot{\gamma } \end{array}\right]=J\left[\begin{array}{c} {\dot{\theta }}_{1}\\ {\dot{\theta }}_{2} \end{array}\right],\;J=\frac{r_{w}}{2R}\;M,\;M=\left[\begin{array}{cc} 1 & 1\\ 1 & -1 \end{array}\right].$$

Common-mode rolling drives the orbital angle, $\dot{\phi }=\frac{r_{w}}{2R}\left({\dot{\theta }}_{1}+{\dot{\theta }}_{2}\right)$, while differential rolling drives the contact angle, $\dot{\gamma }=\frac{r_{w}}{2R}\left({\dot{\theta }}_{1}-{\dot{\theta }}_{2}\right)$: the two lateral contacts slide apart or together along the surface.

Elsewhere in the paper *γ* is treated as a fixed quantity, which requires reconciling with (7) and (8). During grasping, the arms are free to move and *γ* varies with them; once the grasp has closed on a given cylinder, however, the arm angle $\theta_{a}$ is held by the grasping actuator, and with $\theta_{a}$ held the contact geometry of Section 4.2 fixes *γ* as well. A sustained differential torque applied in that state does not, therefore, produce the rolling motion $\dot{\gamma }\neq 0$ that (7) would describe: the wheels do not turn, and the torque is reacted by static friction at the two lateral contacts, in the same way that a torque applied to a blocked wheel produces a tangential contact force without rotation. This is precisely the mechanism by which Δτ modulates the contact normal forces—the effect quantified in (20)—which is what allows the grasp to counteract the orientation-dependent gravitational load of (23) as the vehicle orbits, in particular at the contacts lying below the equator of the cylinder cross-section, where the normal direction has a component opposing gravity. Equations (7) and (8) are used in this paper only to obtain the operational Jacobian whose transpose maps torques to forces by kineto-static duality; the associated motion along *γ* is virtual, in the usual sense of that duality, and need not be realised. The validity of this description is bounded by the Coulomb condition (19): it holds as long as the commanded torques keep each lateral contact inside its friction cone, which is exactly what the bounds of Section 5.3 enforce.

The radial position follows as the dependent output

(9) $$\rho =∥C∥=R+a\left(\theta_{0}\right),\;\frac{d\rho }{d\gamma }>0,$$

since opening the contact angle lifts the platform away from the cylinder axis. Differentiating the contact-closure condition gives the *radial force lever* in closed form,

(10) $$\frac{d\rho }{d\gamma }=\left(R+r_{w}\right)\left(\sin\gamma -\cos\gamma \cot\theta_{a}\right),$$

which is strictly positive over the whole admissible range, decreasing from 1.07 to 0.27 mm/deg as the cylinder shrinks from D=87 to 45 mm.

*Force duality. M* is symmetric with $M^{2}=2I$, so $J^{-⊤}=\frac{R}{r_{w}}M$. By kineto-static duality the wheel torques and the operational wrench $F={\left[\tau_{\phi },\;\tau_{\gamma }\right]}^{⊤}$—conjugate to ${\left[\dot{\phi },\dot{\gamma }\right]}^{⊤}$—are related by $\tau =J^{⊤}F$, whose inverse gives the decomposition directly:

(11) $$\left[\begin{array}{c} \tau_{\phi }\\ \tau_{\gamma } \end{array}\right]=J^{-⊤}\left[\begin{array}{c} \tau_{w_{1}}\\ \tau_{w_{2}} \end{array}\right]=\frac{R}{r_{w}}\left[\begin{array}{c} \tau_{w_{1}}+\tau_{w_{2}}\\ \tau_{w_{1}}-\tau_{w_{2}} \end{array}\right]=\frac{R}{r_{w}}\left[\begin{array}{c} \tau_{\Sigma }\\ \Delta \tau \end{array}\right].$$

Read backwards, (11) is also the *allocation* law used by the controller: since $M^{-1}=M/2$, a desired pair of channel commands $\left(\tau_{\Sigma ,d},\Delta \tau_{d}\right)$ is distributed over the two physical wheels as

(12) $$\left[\begin{array}{c} \tau_{w_{1}}\\ \tau_{w_{2}} \end{array}\right]=\frac{1}{2}M\left[\begin{array}{c} \tau_{\Sigma ,d}\\ \Delta \tau_{d} \end{array}\right]=\frac{1}{2}\left[\begin{array}{c} \tau_{\Sigma ,d}+\Delta \tau_{d}\\ \tau_{\Sigma ,d}-\Delta \tau_{d} \end{array}\right],$$

so the orbital loop and the radial force loop can write to the same two actuators simultaneously (Section 7).

The rows of *M* are orthogonal, so the two channels do not contaminate each other. This is the key structural result of the paper: **equal and opposite lateral wheel torques produce *no* orbital moment; they produce exactly and only the generalised force $\tau_{\gamma }$ conjugate to the contact angle.** And because *γ* is kinematically tied to the radial position through (9) and (10), that force acts along the radial direction: driving the wheels apart (Δτ>0) opens *γ*, lifts the platform (ρ↑), extends the swing links (a↑) and *unloads* the central contact; driving them together closes *γ* and *loads* it. The differential channel is therefore the radial force channel, and it is orthogonal to the orbital channel by construction—the property exploited by the control architecture of Section 7. Section 5 quantifies the resulting gain, $\partial f_{n_{0}}/\partial \Delta \tau =-\sin\gamma /r_{w}$, by a direct force balance at the contacts.

The static force model of Section 5 uses the three Cartesian contact positions $P_{c_{0}}$, $P_{c_{1}}$, and $P_{c_{2}}$ obtained above, which follow from the arm opening $\theta_{a}$ and the measured swing-link deflections $\theta_{0}$, as defined in Section 4.

## 5. Static Force Model

To analyse grasp stability and traction limits during locomotion on cylindrical surfaces, a compact model of the wheel–surface contact forces is required. This section derives a static formulation relating the actuator inputs—namely grasping torque and wheel-drive torques—to the normal and tangential forces at the three contact points. These expressions are subsequently used to establish equilibrium conditions and friction constraints for stable grasping and propulsion.

**Figure 3:**
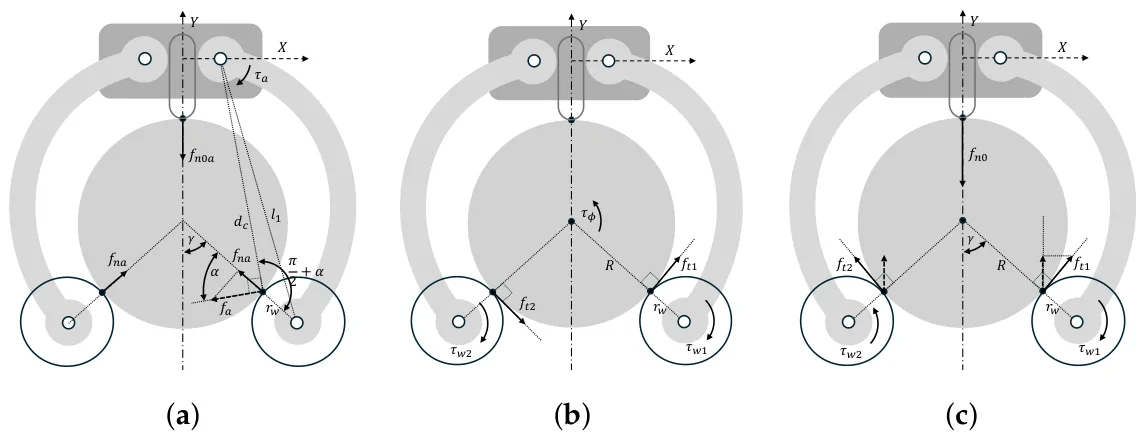
Static force model. (**a**) Isolated effect of the grasping (closing) torque $\tau_{a}$, applied through the central actuator, on the normal contact forces at the three contact points: $f_{n,a}$ at the lateral wheels and $f_{n_{0},a}$ at the central wheel (subscript *a* denotes the force component induced by $\tau_{a}$ alone). (**b**) Isolated effect of same-direction, equal-magnitude lateral wheel torques $\tau_{w}$ on the tangential forces and, consequently, on the net torque driving the vehicle’s rotation about the cylinder axis. (**c**) Isolated effect of opposite-direction, equal-magnitude lateral wheel torques on the tangential forces: the resulting directional difference produces a radial resultant that translates into a normal force at the central contact $P_{c_{0}}$. The combination of (**b**,**c**) is captured by the Jacobian of Section 4 (Equation (11)), which determines the vehicle’s orbital and radial force torque allocation, while the grasping torque $\tau_{a}$ in (**a**) supplies the normal forces required for stable grasping and slip-free wheel rotation.

The model assumes rigid links and wheels, localised point contacts, and Coulomb friction at each wheel–surface interface. Figure 3 summarises the three elementary actuation effects analysed in this section.

These assumptions bound the regime in which the resulting expressions apply, in two respects. First, the distal wheels of the prototype are omnidirectional, so their friction capability is anisotropic: traction is developed along the driven direction, while the passive rollers offer comparatively little resistance transverse to it. Representing each contact by a single isotropic coefficient *μ* is therefore a simplification, admissible here because the tangential forces that the model resolves—those produced by the wheel torques through (18)—act along the driven direction, which is also the direction of the rolling constraint (7). The transverse direction carries no commanded force in this architecture, but it also provides little resistance to unmodelled transverse disturbances, and the value *μ* = 0.5 used throughout should be read as an effective coefficient along the driven direction rather than as a property of the wheel–surface pair in general. Second, the contacts are treated as rigid and localised. On a compliant substrate such as skin, the contact would instead be a finite patch with viscoelastic behaviour, introducing rate-dependent effects and shear dragging that this static formulation does not represent, and that would modify both the effective moment arms and the slip condition. The tests reported in Section 8.4 use a rigid cylinder, so this limitation is not exercised there; characterising the contact mechanics on soft tissue, and extending the model accordingly, is required before the approach can be applied on a human limb.

### 5.1. Forces Caused by the Grasping Actuator

The purpose of this section is to determine the *minimum grasping torque* $\tau_{a}$ that the actuator must deliver for a stable grasp. To that end we first establish how $\tau_{a}$ maps onto the normal force at each lateral contact—i.e., how strongly the mechanism squeezes the object—and then invert that map. A static contact model is considered first, neglecting both friction and gravity, so the normal force balance is written as

(13) $$\sum_{i\in \{0,1,2\}}{\vec{F}}_{i}=0,\;F_{n,i}\geq 0,\;F_{n,i}\geq f_{\mathrm{slip}}\;\mathrm{for}\;i\in \left\{1,2\right\}.$$

The Coulomb bound $f_{\mathrm{slip}}$ applies only to the two *lateral* contacts, which carry the drive torques; the central contact bears no tangential load ($f_{t_{0}}\approx 0$) and is subject only to non-detachment. Its explicit form, derived in Section 5.3 once gravity is included, is (25b). With gravity neglected here, the grasping torque is shared equally between the two lateral contacts.

Therefore, each lateral contact is driven by half of the grasping torque. Consider first the *isolated* effect of the actuator, as depicted in Figure 3a: if the lateral wheel was locked, the closing torque would deliver at the contact an interaction force of magnitude

(14) $$f_{a}=\frac{\tau_{a}}{2\;d_{c}},\;d_{c}\left(\theta_{a}\right)=∥P_{c_{1}}\left(\theta_{a}\right)-P_{s}∥,$$

where $d_{c}$ is the *contact distance*, i.e., the moment arm of the grasping actuator: the distance from the actuator axis $P_{s}=\left(l_{0},0\right)$ to the wheel–cylinder contact point. Unlike the link lengths of Table 1, $d_{c}$ is not a design constant but a configuration-dependent quantity, recomputed at every sample from the contact geometry of Section 4, since $P_{c_{1}}$ migrates over the wheel as the arm opens and as the cylinder diameter changes.

The force $f_{a}$ acts perpendicular to the lever arm, along the unit vector

(15) $$e_{a}=\frac{1}{d_{c}}\left(l_{y},\;-l_{x}\right),\;l=P_{c_{1}}-P_{s},$$

which is in general *not* aligned with the contact normal $n_{i}$. Only the projection of $f_{a}$ onto $n_{i}$ presses the wheel against the surface. Writing *α* for the angle between $e_{a}$ and $n_{i}$, the normal force induced by the actuator alone is

(16) $$f_{n,a}=f_{a}\;\left(e_{a}\cdot n_{i}\right)=\frac{\tau_{a}}{2\;d_{c}}\cos\alpha .$$

The complementary component $f_{a}\sin\alpha$ lies along $t_{i}$ and is *not* sustained by the arm: the tangential force at a lateral contact is set by that wheel’s own torque (Section 5.2), not by the grasping actuator. This is precisely why Figure 3 separates the three elementary effects. Because the gear coupling described in Section 3 constrains both arms to the same magnitude of rotation, the two-finger configuration is symmetric by construction, so the normal force vector is $F_{n,i}=f_{n,i}n_{i}$ with equal load sharing $f_{n_{1}}=f_{n_{2}}=f_{n,a}$.

Inverting (16) finally gives the grasping torque required to sustain a prescribed lateral normal force, which is the result this section set out to obtain:

(17) $$\tau_{a}=\frac{2\;d_{c}\left(\theta_{a}\right)}{\cos\alpha }\;f_{n_{1}}$$

The *minimum* admissible torque, $\tau_{a}^{\min}\left(\phi \right)$, is obtained by substituting into (17) the largest lateral normal force demanded by the contact-stability conditions—non-detachment of the central wheel and absence of slip at either lateral wheel—which depend on gravity and are therefore derived in Section 5.3. Both geometric factors work against the actuator on small cylinders: $d_{c}$ shrinks only mildly while cosα collapses from 0.91 to 0.45 over the admissible diameter range, so a given normal force costs up to twice the torque at D=45mm than at D=87mm.

### 5.2. Forces Caused by Wheel’s Torques

For each driven lateral wheel *i*, the applied motor torque $\tau_{w,i}$ produces a tangential contact force

(18) $$f_{t,i}=\frac{\tau_{w,i}}{r_{w}}.$$

The direction of $F_{t,i}$ is the unit tangential vector $t_{i}={\left[\;n_{iy},\;-n_{ix}\right]}^{⊤}$, orthogonal to $n_{i}$ and oriented along increasing arc angle on the cylinder—the same orientation used by the rolling constraint (7), which is what allows the kinematic and static descriptions to share one sign convention. The total contact force decomposes as $F_{i}=f_{n,i}\;n_{i}+f_{t,i}\;t_{i}$, and the admissible tangential force is bounded by Coulomb friction,

(19) $$|f_{t,i}|\leq \mu f_{n,i},\;f_{n,i}\geq 0,$$

where *μ* is the coefficient of static friction.

We now determine how the two wheel torques act on the vehicle. Substituting $t_{1}=\left(\cos\gamma ,\;\sin\gamma \right)$ and $t_{2}=\left(\cos\gamma ,\;-\sin\gamma \right)$ from (5), the resultant of the two tangential contact forces is $F_{t}=f_{t,1}t_{1}+f_{t,2}t_{2}=\frac{1}{r_{w}}{\left(\tau_{\Sigma }\cos\gamma ,\;\Delta \tau \sin\gamma \right)}^{⊤}$, with the two actuation channels $\tau_{\Sigma }=\tau_{w_{1}}+\tau_{w_{2}}$ and $\Delta \tau =\tau_{w_{1}}-\tau_{w_{2}}$ of (11). These are the two elementary cases of Figure 3b,c: same-direction equal torques (Figure 3b) give a resultant *parallel* to the surface at the pole, and opposite equal torques (Figure 3c) one *perpendicular* to it. Taking moments about the cylinder axis and projecting onto the central contact normal $n_{0}$ therefore gives a *diagonal* map:

(20) $$\left[\begin{array}{c} \tau_{\phi }\\ \Delta f_{n_{0}} \end{array}\right]=\left[\begin{array}{cc} \frac{R}{r_{w}} & 0\\ 0 & -\frac{\sin\gamma }{r_{w}} \end{array}\right]\left[\begin{array}{c} \tau_{\Sigma }\\ \Delta \tau \end{array}\right].$$

The two off-diagonal entries are identically zero, not merely small. The mirror symmetry of the contacts makes the tangential projections onto $n_{0}$ equal and opposite, $t_{1}\cdot n_{0}=-t_{2}\cdot n_{0}=-\sin\gamma$ by (6), so equal torques cancel in the radial direction, and their moment arms about the cylinder axis are equal, so opposite torques cancel in the rotational direction. Equation (20) is the static counterpart of the kineto-static duality (11): the same basis $\left\{\tau_{\Sigma },\Delta \tau \right\}$ that diagonalises the velocity Jacobian also diagonalises the force map.

*Robustness of the two zero entries.* The two off-diagonal terms of (20) do not rest on the same assumption. The upper-right entry ($\Delta \tau \to \tau_{\phi }$) is zero for purely geometric reasons that hold even if the two lateral contacts are not mirror-symmetric: a tangential force is by definition perpendicular to the radius at its own contact point, so its moment about the cylinder axis is $Rf_{t,i}$ regardless of that contact’s angular position; summing $\tau_{\phi }=R\left(f_{t,1}+f_{t,2}\right)/1=\left(R/r_{w}\right)\tau_{\Sigma }$ therefore never contains Δτ, for any $\gamma_{1},\gamma_{2}$. The lower-left entry ($\tau_{\Sigma }\to \Delta f_{n_{0}}$), in contrast, does rely on $\gamma_{1}=\gamma_{2}\equiv \gamma$: repeating the projection with independent contact angles $\gamma_{1}\neq \gamma_{2}$ introduces a spurious term $\tau_{\Sigma }\cdot \frac{1}{2}\left(\sin\gamma_{1}-\sin\gamma_{2}\right)$ alongside the intended Δτ dependence. Exact symmetry is not an idealisation assumed for convenience here; however, it is enforced mechanically by the single-actuator gear coupling of Section 3 (Figure 1), which constrains $\theta_{1}=\theta_{2}$ so that $P_{1}$ and $P_{2}$—and hence $\gamma_{1}$ and $\gamma_{2}$—are exact mirror images by construction. The only residual source of asymmetry is backlash in that coupling, $\Delta \gamma_{\mathrm{bl}}$, which propagates to first order as $\Delta f_{n_{0}}^{\mathrm{par}}\approx \tau_{\Sigma }\cos\gamma \;\Delta \gamma_{\mathrm{bl}}/\left(2r_{w}\right)$. Taking the manufacturer-specified backlash of the grasping servomotor used in the prototype (Section 8.4) as a partial bound—15–25 arcmin, i.e., $0.25^{\circ }$–$0.45^{\circ }$, a range typical of geared servos of this class—this parasitic coupling stays below 1% of the differential channel’s designed authority $\left(\sin\gamma /r_{w}\right)\Delta \tau_{\max}$ over the tested operating range; the 3D-printed gear train that couples the two arms may add further, uncharacterised backlash, but the coupling grows only linearly with it, and a combined backlash in excess of $5^{\circ }$—roughly an order of magnitude above typical values for a printed coupling of this size—would be required to reach 10% of that authority.

Two consequences follow, and they are the basis of the whole control architecture of Section 7:

- **Rotation** (Figure 3b). The torque driving the vehicle around the cylinder is set *only* by the common-mode component, $\tau_{\phi }=\left(R/r_{w}\right)\;\tau_{\Sigma }$. A purely differential command ($\tau_{w_{1}}=-\tau_{w_{2}}$) produces no orbital torque at all.
- **Central normal force** (Figure 3c). The central contact load is modulated *only* by the differential component, $\Delta f_{n_{0}}=-\left(\sin\gamma /r_{w}\right)\;\Delta \tau$. A purely common-mode command leaves $f_{n_{0}}$ untouched. The gain $\sin\gamma /r_{w}$ is the geometric authority of this channel, and it collapses as γ→0: on the smallest admissible cylinder ($\gamma =27^{\circ }$) the differential channel is roughly half as effective as on the largest ($\gamma \to 90^{\circ }$).

### 5.3. Effects of Gravity and Friction on the Contact Forces

**Figure 4:**
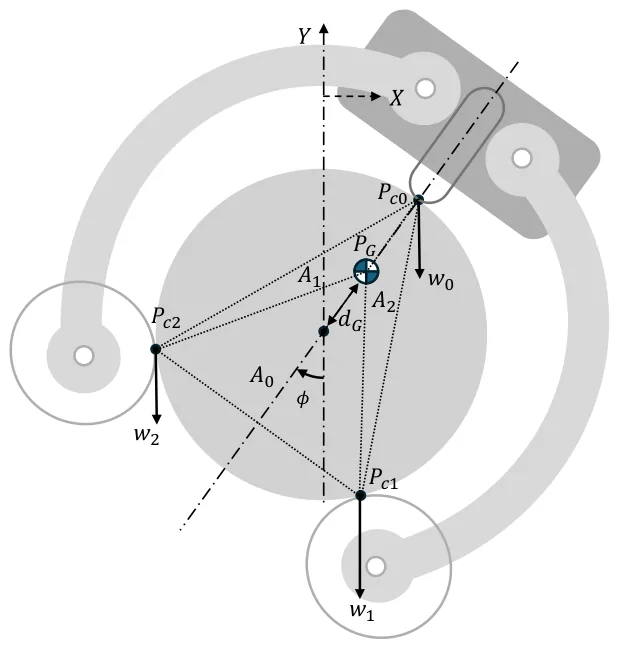
Support triangle and barycentric load distribution for the three-contact model. $P_{c0}$, $P_{c1}$, and $P_{c2}$ represent the three contact points, while $P_{G}$ is the projection of the center of mass (CoM) onto the contact plane. $A_{0}$, $A_{1}$, and $A_{2}$ are the barycentric subtriangle areas, and $w_{0}$, $w_{1}$, and $w_{2}$ the corresponding vertical loads.

Gravity introduces an external load that modifies both the normal and tangential components $n_{i}$ and $t_{i}$, required to maintain static equilibrium. In the present model, the robot weight W=m·g is distributed among the three contact points according to the projection of the centre of mass (*CoM*) onto the contact plane (see Figure 4). Let $P_{G}$ denote the projection of the *CoM*. For all three contacts to sustain compressive loads, $P_{G}$ must lie within the support triangle defined by $P_{c_{0}}$,$P_{c_{1}}$ and $P_{c_{2}}$. This condition is written as

(21) $$P_{G}\in \mathrm{conv}P_{c_{0}},P_{c_{1}},P_{c_{2}}\;\Leftrightarrow \;w_{i}\geq 0,\;\forall i,$$

which guarantees compressive normal reactions at all contacts. If $P_{G}$ exits the support triangle, some $w_{k}<0$, meaning contact *k* cannot sustain the load and lifts off; the system then reduces to two supporting contacts and may tip about the nearest edge.

Therefore, the three-contact model is applied when $P_{G}$ remains inside the support triangle; otherwise, the force distribution must enforce contact and friction constraints. The vertical load at each contact, $w_{i}$, is then computed using barycentric (area) coordinates,

(22) $$w_{i}=W\;\frac{A_{i}}{A},\;i\in \left\{0,1,2\right\},$$

where *A* is the area of the support triangle and $A_{i}$ is the area of the subtriangle formed by replacing vertex *i* with the projected CoM. By construction, $\sum_{i}w_{i}=W$ and $w_{i}\geq 0$ when the CoM projection lies within the support polygon. The scope of (22) is limited in two ways. First, on the present geometry the applicability condition $P_{G}\in \mathrm{conv}\left\{P_{c_{0}},P_{c_{1}},P_{c_{2}}\right\}$ reduces to a simple inequality: since $P_{G}$ and $P_{c_{0}}$ and the cylinder axis are collinear along the vehicle’s central axis, and $P_{c_{0}}$ is the vertex of the triangle closest to that axis, $P_{G}$ lies inside the triangle if and only if $d_{G}\leq R$. Whether this holds is a matter of mass distribution (battery and electronics placement) rather than of the kinematic design, and it is not fixed in the present prototype. Second, and independently of that condition, the $w_{i}$ of (22) enter the contact and slip conditions (25) that set the required grasping torque $\tau_{a}$; they are not used to evaluate the central normal force $f_{n_{0}}$, which follows instead from the force balance of Section 5.4. The two quantities are inputs to different blocks of the control architecture of Section 7, not alternative estimates of the same load.

Let $\hat{y}$ denote the unit vector along the gravity direction in the plane. The gravity contribution at contact *i* is $w_{i}\hat{y}$, whose projections onto the local basis yield the gravity-compensated components

(23) $$f_{n,i}^{\prime }=f_{n,i}-w_{i}\left(\hat{y}\cdot n_{i}\right),$$

(24) $$f_{t,i}^{\prime }=f_{t,i}-w_{i}\left(\hat{y}\cdot t_{i}\right).$$

Equations (23) and (24) show that gravity can either increase or decrease the normal loading depending on contact orientation, while simultaneously imposing a tangential bias that must be counteracted by traction to prevent slip.

The conditions derived above define a complete set of inequality constraints that must hold simultaneously for stable orbital grasping. Collecting the key bounds, the admissible actuation set $\tau \left(\phi \right)={\left[\tau_{a}\left(\phi \right),\tau_{w_{1}}\left(\phi \right),\tau_{w_{2}}\left(\phi \right)\right]}^{⊤}$ must satisfy

(25a) $$\begin{array}{cc} f_{n,0}\left(\phi ,\tau_{w_{1}},\tau_{w_{2}}\right) & \geq 0,\; \end{array}$$

(25b) $$\begin{array}{cc} F_{n,i}^{\mathrm{net}}\left(\phi \right) & \geq f_{\mathrm{slip}}\left(\phi \right)=\frac{|\tau_{g}\left(\phi \right)|}{2\mu R},\;i\in \left\{1,2\right\},\; \end{array}$$

(25c) $$\begin{array}{cc} |\tau_{w,i}\left(\phi \right)| & \leq \mu \;f_{n,i}\left(\phi \right)\;r_{w},\;i\in \left\{1,2\right\},\; \end{array}$$

(25d) $$\begin{array}{cc} \;\tau_{a}\left(\phi \right) & \geq \max_{i\in \{1,2\}}\;\left(\frac{2d_{c}\left(\theta_{a}\right)}{\cos\alpha }\;\left[\frac{|\tau_{g}\left(\phi \right)|}{2\mu R}-n_{i}^{⊤}\left(\phi \right)F_{g}\left(\phi \right)\right]\right). \end{array}$$

Constraint (25a) ensures the central traction module remains in contact with the surface (non-detachment). Constraint (25b) guarantees that each lateral contact carries sufficient normal preload to prevent slip under the gravity-induced tangential bias. Constraint (25c) enforces the Coulomb friction limit, bounding the admissible traction torque for a given normal preload. Finally, (25d) gives the orientation-dependent lower bound on the grasping torque that simultaneously prevents lift-off and wheel slip over the full $360^{\circ }$ orbital range. The satisfaction of (25) at every *ϕ* is the necessary and sufficient condition for contact-consistent locomotion under the static model.

### 5.4. Superposition: The Combined Central Contact Force

The three elementary effects analysed above—the grasping actuator, the wheel torques and gravity—act on the same three contacts, so it remains to collect them into a single expression. Because the static model is linear in the applied forces, they superpose. Static equilibrium of the vehicle reads

(26) $$F_{0}+F_{1}+F_{2}+W\left(\phi \right)=0,$$

where $F_{i}$ is the contact force exerted *on the vehicle* at contact *i* and W(ϕ) is the weight. Assuming the central wheel generates no traction of its own ($f_{t_{0}}\approx 0$), so that $F_{0}=-f_{n_{0}}n_{0}$, and projecting (26) onto $n_{0}$, using the geometric projections of (6) and the actuator model (16), gives the central normal force as the sum of three independent contributions:

(27) $$f_{n_{0}}\left(\phi ,\tau_{a},\Delta \tau \right)=\underset{\mathrm{gravity}}{\underset{︸}{mg\cos\phi }}\;+\;\underset{\mathrm{grasping}\;\mathrm{actuator}}{\underset{︸}{\frac{\cos\alpha \;\cos\gamma }{d_{c}\left(\theta_{a}\right)}\;\tau_{a}}}\;-\;\underset{\mathrm{differential}\;\mathrm{traction}}{\underset{︸}{\frac{\sin\gamma }{r_{w}}\;\Delta \tau }}.$$

Equation (27) is the projection of the vector balance (26) onto the single direction $n_{0}$, evaluated with $f_{n_{1}}$ and $f_{n_{2}}$ already fixed by the grasping actuator through (16) and $f_{t_{1}}$ and $f_{t_{2}}$ by the wheel torques through (18). It therefore does not require, and does not use, the area-based load split of (22): the full weight resultant is equilibrated by the three contacts acting together, and mgcosϕ is the component of that resultant along $n_{0}$, which is the direction the central contact must react. The remaining components of (26), and the moment balance, are carried by the arm pivots and the rigid chassis, which are internal to the vehicle and therefore do not enter the contact force expression sought here.

Three properties of (27) organise the rest of the paper.

First, $f_{n_{0}}$ is *affine* in both commanded torques, with purely geometric gains that the estimator of Section 4 supplies online. This is what makes an exact feedforward inversion possible (Section 7.2) rather than a switching law.

Second, the common-mode torque $\tau_{\Sigma }$ *does not appear at all*. Orbital propulsion is therefore invisible to the central contact: the robot can accelerate, decelerate or hold station around the cylinder without perturbing its own grip. Conversely, by (20), Δτ produces no orbital torque. The two channels are exactly, not approximately, decoupled.

Third, the two remaining terms have opposite roles. The baseline

(28) $$f_{n_{0},0}\left(\phi ,\tau_{a}\right)=mg\cos\phi +\frac{\cos\alpha \;\cos\gamma }{d_{c}\left(\theta_{a}\right)}\;\tau_{a}$$

is set by the grasp and by orientation, and is *not* freely available for regulation: $\tau_{a}$ is already pinned by the stability bound (25d), and the gravity term swings by 2mg over one revolution, vanishing at $\phi =90^{\circ }$ and $270^{\circ }$ where the weight becomes purely tangential. The differential term $-(\sin\gamma /r_{w})\Delta \tau$ is what remains free, and it is precisely the actuator authority used to hold $f_{n_{0}}$ inside the admissible band $\left(f_{n_{0},\min},\;f_{n_{0},\max}\right)$ against that swing. Increasing Δτ lowers $f_{n_{0}}$ (self-opening); decreasing it raises $f_{n_{0}}$ (self-closing); neither disturbs the orbital motion. This is the coupling exploited by the radial force controller of Section 7.2.

## 6. Orbital Dynamics

This section has two goals: to write the scalar equation of motion for the orbital rotation, identifying the disturbance that the orbital controller must reject, and to bound the worst-case grasping preload that keeps the wheels slip-free during that motion. The system is modelled as a 1-DOF rotational mechanism: the mobile platform (MP) rotates about the fixed longitudinal axis of the cylinder, parametrised by the orbital angle *ϕ*, with all three wheels rolling on the surface and the grasp configuration $\theta_{a}$ treated as quasi-static. Only the terms needed for these two goals are modelled in detail; the lumped inertia and rolling-resistance coefficients are not identified individually, as they are rejected by the velocity loop rather than compensated in feedforward.

### 6.1. Orbital Equation of Motion

Applying Newton–Euler mechanics about the cylinder’s longitudinal axis yields the scalar equation of motion

(29) $$I_{zz}\;\ddot{\phi }+\tau_{g}\left(\phi \right)+\tau_{f}\left(\dot{\phi }\right)=\tau_{\phi ,\mathrm{act}},$$

where each term is described below.

*Inertial term.* The moment of inertia $I_{zz}$ about the cylinder’s axis is approximated as a lumped parameter $I_{zz}\approx m\;d_{G}^{2}$, where *m* is the total vehicle mass and $d_{G}$ is the distance from the cylinder axis to the vehicle centre of mass. This approximation is sufficient for the quasi-static stability analysis and for bounding worst-case inertial loads during transients; it is not used directly in the gravity-feedforward controller.

*Gravitational bias torque.* The gravitational torque $\tau_{g}\left(\phi \right)$ represents the component of the vehicle’s weight that acts tangentially to the orbit, tending to roll the robot toward the bottom of the cylinder. With $\hat{g}={\left[0,-g\right]}^{⊤}$ and the robot CoM at distance $d_{G}$ from the cylinder axis,

(30) $$\tau_{g}\left(\phi \right)=-m\;g\;d_{G}\sin\left(\phi \right),$$

where $m=m_{0}+2m_{a}+4m_{w}$ is the total vehicle mass and sin(ϕ)=0 corresponds to the gravity-aligned (top or bottom) equilibrium. This sinusoidal bias is the primary disturbance that both the grasping and traction controllers must counteract.

*Friction torque.* The friction torque $\tau_{f}\left(\dot{\phi }\right)$ aggregates viscous and dry (Coulomb) rolling resistance at all wheel–cylinder contacts,

(31) $$\tau_{f}\left(\dot{\phi }\right)=b_{v}\;\dot{\phi }+\sum_{i\in \{0,1,2\}}f_{n,i}\left(\phi \right)\;c_{r,i}\;R_{i}\;\mathrm{sgn}\left(\dot{\phi }\right),$$

where $b_{v}$ is the lumped viscous damping coefficient, $c_{r,i}$ is the rolling-resistance coefficient at contact *i*, and $R_{i}$ is the corresponding orbit radius. For the skin–polyurethane interface, rolling resistance is small relative to gravitational and inertial terms but is included to bound the minimum traction torque required for sustained motion.

*Actuation torque.* The net driving torque is supplied entirely by the common-mode channel, as established in (20):

(32) $$\tau_{\phi ,\mathrm{act}}=\frac{R}{r_{w}}\;\tau_{\Sigma },\;\tau_{\Sigma }=\tau_{w_{1}}+\tau_{w_{2}}.$$

The differential channel contributes nothing here; it is reserved for regulating the central contact force, and it does so without disturbing (29).

### 6.2. Dynamic Stability Conditions

The instantaneous traction demand is $\tau_{\phi ,\mathrm{act}}=I_{zz}\ddot{\phi }+\tau_{g}\left(\phi \right)+\tau_{f}\left(\dot{\phi }\right)$ from (29). Of these, only the gravitational bias $\tau_{g}\left(\phi \right)$ is cancelled in feedforward by the orbital controller (Section 7.1); the inertial and friction terms are rejected by the PI velocity loop, which justifies focussing the feedforward design on gravity compensation alone. For bounding the worst-case grasping preload, however, all three terms enter through the common-mode torque $\tau_{\Sigma }=\left(r_{w}/R\right)\;\tau_{\phi ,\mathrm{act}}$, giving the minimum slip-free lateral preload

(33) $$f_{n_{1}}\left(\phi \right)\geq \frac{|\tau_{\Sigma }\left(\phi \right)|}{2\mu r_{w}}=\frac{|I_{zz}\ddot{\phi }+\tau_{g}\left(\phi \right)+\tau_{f}\left(\dot{\phi }\right)|}{2\mu R}.$$

In the quasi-static limit ($\ddot{\phi }\to 0$, $\tau_{f}\to 0$) this reduces to the static slip threshold (25b); the inertial correction $I_{zz}\ddot{\phi }/R$ matters only during acceleration transients and sets the worst-case preload the grasp must accommodate.

## 7. Control Architecture

**Figure 5:**
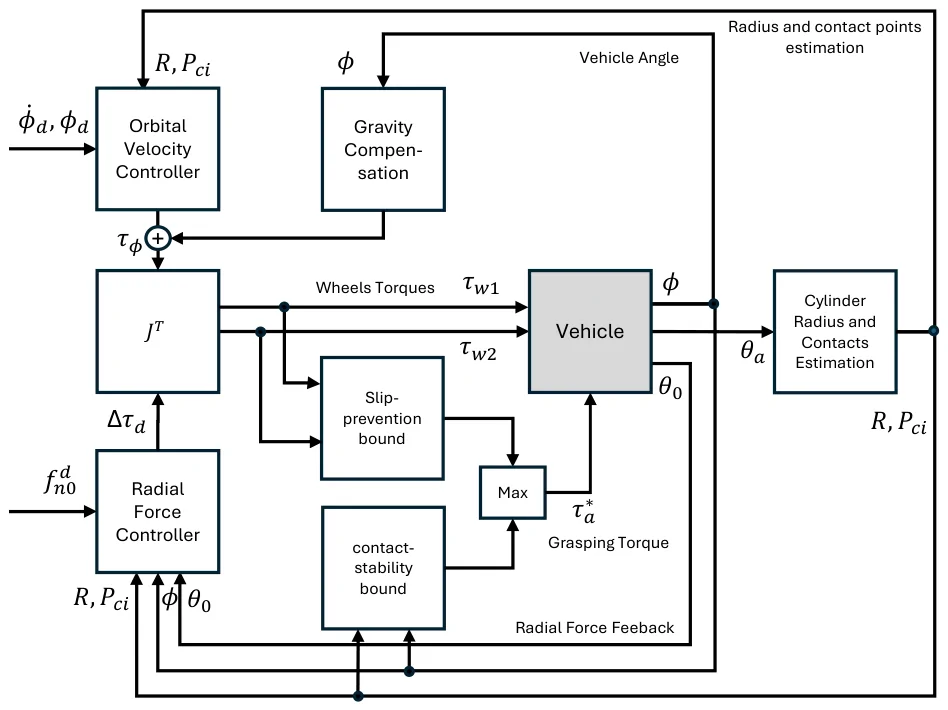
Block diagram of the proposed control architecture. The orbital velocity controller (with a separate gravity compensation feedforward) and the radial force controller produce the orbital moment $\tau_{\phi }$ and the differential command $\Delta \tau_{d}$, respectively, which are mapped to the wheel torques $\tau_{w_{1}}$, $\tau_{w_{2}}$ by the allocation block $J^{⊤}$ (34). The minimum grasping force block computes $\tau_{a}^{*}$ as the maximum of a contact-stability bound (stable grasping) and a slip-prevention bound (slippage avoidance). The $\theta_{0}$ feedback path closes each loop: it provides the single central force estimate ${\hat{f}}_{n_{0}}$ from the spring calibration and the mean contact depth $a(\theta_{0})$ to the estimator. The cylinder radius and contact estimation block supplies *R* and $P_{ci}$ from $\theta_{a}$ and $\theta_{0}$ to all blocks in real time.

Figure 5 shows the overall block diagram. The architecture has four functional blocks whose outputs converge through the actuation Jacobian transpose $J^{⊤}$ onto the two lateral wheel torques $\tau_{w_{1}}$ and $\tau_{w_{2}}$ and the grasping torque $\tau_{a}^{*}$.

The **orbital velocity controller** (Section 7.1) receives the desired velocity ${\dot{\phi }}^{d}$, the orbital angle *ϕ*, and the estimated radius *R* and contact points $P_{ci}$. Its PI output is added to the feedforward term of a separate **gravity compensation** block, which reads the current angle *ϕ*; the sum is the orbital moment $\tau_{\phi }$.

The **radial force controller** (Section 7.2) receives the desired central force $f_{n_{0}}^{d}$ (labelled $f_{\rho d}$ in Figure 5), together with *R*, $P_{ci}$, *ϕ*, and the central contact measurement $\theta_{0}$, and outputs the differential torque command $\Delta \tau_{d}$ that regulates it (the arrow labelled $f_{\rho }$ in the figure).

These two operational-space commands are mapped to wheel torques by the allocation block labelled $J^{⊤}$ in Figure 5, which is the transpose of the velocity Jacobian and, equivalently, the inverse of the actuation map (12),

(34) $$\left[\begin{array}{c} \tau_{w_{1}}\\ \tau_{w_{2}} \end{array}\right]=\frac{1}{2}\left[\begin{array}{c} \tau_{\Sigma ,d}+\Delta \tau_{d}\\ \tau_{\Sigma ,d}-\Delta \tau_{d} \end{array}\right],\;\tau_{\Sigma ,d}=\frac{\tau_{\phi }}{\kappa },\;\kappa =\frac{R}{r_{w}},$$

where $\tau_{\phi }$ is the orbital moment from the velocity controller and $\Delta \tau_{d}$ the differential torque from the radial force controller (Section 7.2).

The **minimum grasping force** block (Section 7.3) operates in parallel: it receives *ϕ*, $\theta_{0}$, and the current $\tau_{w_{1}}$ and $\tau_{w_{2}}$, and outputs $\tau_{a}^{*}$ as the maximum of a contact-stability bound (stable grasping) and a slip-prevention bound (slippage avoidance). The coefficient *μ* is a shared model parameter that enters both bounds and the Coulomb saturation of $\tau_{\phi }$.

A **force feedback** path closes the measured swing-link deflections $\theta_{0}$ into all three blocks: through the spring calibration (46) it supplies the single central force estimate ${\hat{f}}_{n_{0}}$ to the radial force loop, and through (1) it supplies the current contact depth $a(\theta_{0})$ to the radius and contact estimator. It is drawn dashed in Figure 5 because the architecture degrades gracefully to pure feedforward on a variant without the compliant central mechanism.

The **cylinder radius and contact estimation** block (Section 4.3) runs at every sample from $\theta_{a}$ and $\theta_{0}$, and feeds *R* and $P_{ci}$ back to all control blocks. The following subsections detail each element.

### 7.1. Orbital Velocity Control with Gravity Feedforward

This block tracks a desired orbital velocity profile ${\dot{\phi }}^{d}\left(t\right)$ while compensating for the orientation-dependent gravitational bias $\tau_{g}\left(\phi \right)$. A PI controller on the velocity error $e_{\dot{\phi }}={\dot{\phi }}^{d}-\dot{\phi }$ is combined with a gravity feedforward term to generate the required common-mode torque:

(35) $$\tau_{\Sigma }\left(\phi \right)=\frac{r_{w}}{R}\;\left[\tau_{g}\left(\phi \right)+K_{p}\;e_{\dot{\phi }}+K_{i}\;\int_{0}^{t}\;e_{\dot{\phi }}\;d\sigma \right].$$

The feedforward term $\tau_{g}\left(\phi \right)$ cancels the sinusoidal gravity-induced rolling bias at every orbital angle, so the PI only needs to correct residual velocity errors due to modelling inaccuracies and friction. The resulting common-mode command is split equally between the two wheels, $\tau_{w_{1}}^{\mathrm{ff}}=\tau_{w_{2}}^{\mathrm{ff}}=\tau_{\Sigma }/2$, and is combined with the differential command through the allocation (34). Before being sent to the motor drives, it is saturated to the instantaneous Coulomb friction limit,

(36) $$|\tau_{\Sigma }|\leq 2\;\mu \;f_{n_{1}}\left(\phi \right)\;r_{w},$$

where $f_{n_{1}}\left(\phi \right)$ is supplied in real time by the grasping force computation block (Section 7.3).

### 7.2. Radial Adhesion Force Control

This block regulates the central contact force $f_{n_{0}}$ to a target $f_{n_{0}}^{d}$ inside the admissible band $\left(f_{n_{0},\min},\;f_{n_{0},\max}\right)$ of Section 5.4. The target is not placed at the geometric midpoint of the admissible band but within the differential channel’s authority, $\pm \left(\sin\gamma /r_{w}\right)\;\Delta \tau_{\max}$, about the naturally occurring baseline, so that $\Delta \tau_{d}$ regulates $f_{n_{0}}$ from both sides over one revolution—reducing the grasping force $f_{n_{1}}$ where gravity already presses the central wheel and increasing it half a turn later—while the grasping torque $\tau_{a}$ stays at its minimum wherever possible. Both bounds are set by the compliant sensing mechanism rather than by a material yield limit. The lower bound $f_{n_{0},\min}\approx 1.0\;N$ is *not* a slip threshold: the central wheel carries no drive torque ($f_{t_{0}}\approx 0$, Section 5.4), so its contact bears no tangential load and cannot slip in the Coulomb sense. It is instead a measurement floor—below the spring preload the deflection no longer resolves the load—which conveniently doubles as a contact-persistence (non-detachment) margin above the strict $f_{n_{0}}\geq 0$ limit. The upper bound $f_{n_{0},\max}=6.0\;N$ is the operating ceiling, set conservatively well below the deflection limit of the springs (Section 8.1) so that the estimate stays in the well-resolved part of the characteristic and the pressure on the surface remains modest. Slip is a concern only at the two *lateral* contacts, where the drive torques act; that condition is $f_{n,i}\geq f_{\mathrm{slip}}\left(\phi \right)$ from (25b) and is enforced separately by the grasping-torque law of Section 7.3. The control input is the differential wheel torque $\Delta \tau =\tau_{w_{1}}-\tau_{w_{2}}$, which by the orthogonality of the actuation Jacobian (11) lies in the null space of the orbital motion and therefore does not perturb $\tau_{\Sigma }$ (Section 5.4).

*Feedforward radial force command.* The baseline central force in the absence of any radial force actuation is

(37) $$f_{n_{0},0}\left(\phi \right)=mg\cos\phi +2f_{n_{1}}\left(\phi \right)\cos\gamma .$$

Inverting (27) through the differential channel gives the feedforward radial force command directly as a differential torque,

(38) $$\Delta \tau_{d,\mathrm{ff}}\left(\phi \right)=-\frac{r_{w}}{\sin\gamma }\left(f_{n_{0}}^{d}-f_{n_{0},0}\left(\phi \right)\right),$$

saturated to $\left|\Delta \tau_{d,\mathrm{ff}}\right|\leq \Delta \tau_{\max}$ to respect actuator limits. The gain $r_{w}/\sin\gamma$ is constant for a given grasp, since *γ* is fixed once the arms have closed. This command requires no sensing beyond *ϕ*, $f_{n_{1}}$ and *γ*, all already available from the orbital, grasping and estimation loops, and is always active.

*PI correction from the compliant central contact.* The spring-loaded swing links of the central traction module provide a direct measurement of the central normal force, $f_{n_{0}}^{\mathrm{meas}}={\hat{f}}_{n_{0}}$, obtained from the two swing-link deflections through the spring calibration (46). This measurement closes the radial force loop with an additive PI term that corrects for residual error due to unmodelled friction, geometric tolerances, or coupling-coefficient uncertainty:

(39) $$\Delta \tau_{d,\mathrm{PI}}\left(t\right)=K_{p}^{\left(0\right)}\;e_{f_{n_{0}}}\left(t\right)+K_{i}^{\left(0\right)}\;\int_{0}^{t}\;e_{f_{n_{0}}}\left(\sigma \right)\;d\sigma ,\;e_{f_{n_{0}}}=f_{n_{0}}^{d}-f_{n_{0}}^{\mathrm{meas}}.$$

The total radial force command is

(40) $$\Delta \tau_{d}\left(t\right)=\Delta \tau_{d,\mathrm{ff}}\left(\phi \right)+{⊮}_{\mathrm{sensor}}\cdot \Delta \tau_{d,\mathrm{PI}}\left(t\right),$$

where ${⊮}_{\mathrm{sensor}}\in \left\{0,1\right\}$ indicates sensor availability. The structure is deliberately kept general: on a rigid-central-wheel variant of the platform the term vanishes and $\Delta \tau_{d}=\Delta \tau_{d,\mathrm{ff}}\left(\phi \right)$ alone suffices under nominal conditions. In the prototype reported here ${⊮}_{\mathrm{sensor}}=1$, because the compliant swing links act as the force sensor, so the PI is always active and compensates the systematic deviations that the open-loop feedforward cannot correct. The actuator saturation $\left|\Delta \tau_{d}\right|\leq \Delta \tau_{\max}$ is enforced at the drive level.

The differential torque $\Delta \tau_{d}$ is passed to $J^{⊤}$ (34), which allocates it with opposite signs to the two wheels. The opposite signs guarantee that $\Delta \tau_{d}$ cancels in the common-mode sum $\tau_{\Sigma }=\tau_{w_{1}}+\tau_{w_{2}}$, leaving orbital motion exactly undisturbed. Positive $\Delta \tau_{d}$ decreases $f_{n_{0}}$ (self-opening); negative $\Delta \tau_{d}$ increases it (self-closing), per (27).

### 7.3. Minimum Grasping Force Computation

This block computes the feedforward grasping torque $\tau_{a}^{*}\left(\phi \right)$ through two cascaded stages. The first ensures that all contact normal forces remain strictly positive (no detachment); the second ensures that the lateral normal preload is sufficient to sustain the wheel tangential forces without slip.

*Stage 1—Contact stability (no detachment).*

From the stability analysis of Section 5, the orientation-dependent lower bound that prevents lift-off at all three contacts is

(41) $$\tau_{a}^{\mathrm{stab}}\left(\phi \right)=\max_{i\in \{1,2\}}\;\left(\frac{2d_{c}\left(\theta_{a}\right)}{\cos\alpha }\;\left[\frac{|\tau_{g}\left(\phi \right)|}{2\mu R}-n_{i}^{⊤}\left(\phi \right)F_{g}\left(\phi \right)\right]\right).$$

This depends only on *ϕ* and the identified model parameters; no additional sensing is required.

*Stage 2—Slip prevention (Coulomb constraint).*

Given the wheel torques $\tau_{w_{1}}$ and $\tau_{w_{2}}$ already computed by the orbital and radial force loops, the Coulomb friction condition at each lateral contact requires

(42) $$f_{n_{1}}\geq \frac{|\tau_{w_{i}}|}{\mu \;r_{w}},\;i\in \left\{1,2\right\},$$

which, through (16), translates into a second lower bound on the grasping torque

(43) $$\tau_{a}^{\mathrm{slip}}\left(\phi \right)=\frac{2d_{c}\;\max\;\left(|\tau_{w_{1}}\left|,\;\right|\tau_{w_{2}}|\right)}{\mu \;r_{w}\cos\alpha },$$

with $\tau_{w_{1,2}}=\frac{1}{2}\left(\tau_{\Sigma }\pm \Delta \tau \right)$. The maximum must be evaluated explicitly: the convenient identity $\max(|\tau_{w_{1}}\left|,\right|\tau_{w_{2}}\left|\right)=\frac{1}{2}\left(\right|\tau_{\Sigma }\left|+|\Delta \tau |\right)$ holds only when $\tau_{\Sigma }$ and Δτ have the same sign, and Δτ reverses sign over the revolution by design (Section 7.2), so that identity would size the preload against the less-loaded wheel over part of the orbit. A further subtlety is that (43) and the differential command are mutually dependent, since $\tau_{a}$ sets $f_{n_{1}}$, which shifts the central force baseline (37) and hence Δτ. In the implementation this coupling is resolved conservatively by bounding the differential term with its saturation limit, $\left|\Delta \tau \right|\leq \Delta \tau_{\max}$, which removes the circular dependence at the cost of a slightly larger commanded preload.

*Combined command.*

The final grasping torque is the maximum of both bounds and a constant floor:

(44) $$\tau_{a}^{*}\left(\phi \right)=\max\left(\tau_{a}^{\mathrm{stab}}\left(\phi \right),\;\tau_{a}^{\mathrm{slip}}\left(\phi \right),\;\tau_{a,\min}\right).$$

The floor $\tau_{a,\min}>0$ keeps the grasp closed where the two derived bounds vanish. Both $\tau_{a}^{\mathrm{stab}}$ and $\tau_{a}^{\mathrm{slip}}$ scale with the gravitational load, so both collapse to zero at $\phi =0^{\circ }$ and $180^{\circ }$, where the weight is purely radial and no tangential force must be resisted. Releasing the arms there would satisfy every derived condition while losing the enclosure altogether, leaving the robot unable to react to any disturbance and unable to re-establish contact once the wheels separate from the surface. Enforcing $\tau_{a,\min}$ guarantees a permanent preload, so contact—and therefore the proprioceptive measurement of Section 4—is never interrupted.

This is the engineering counterpart of the safety margin observed in arboreal gripping, where the normal force is held above the strict no-slip minimum rather than at it [4]. For the same reason the commanded torque is applied with a margin over (44) rather than exactly at the bound: the derived expressions supply precisely the friction needed to balance gravity in quasi-static conditions, leaving no reserve for the tangential force required to accelerate, so tracking a trajectory with a grasp set exactly at the bound would slip at every velocity transient. The resulting $\tau_{a}^{*}$ sets $f_{n_{1}}$ at the lateral contacts, which enters both the Coulomb saturation bound (36) for the orbital loop and the central force baseline (37) for the radial force loop. Because $\tau_{a}^{\mathrm{slip}}$ depends on the current wheel torques, this stage introduces a real-time coupling between the grasping and orbital/radial force blocks: tighter wheel commands automatically demand a higher preload, and the grasping actuator responds in the same control cycle. The surface friction coefficient enters both (41) and (43) as a fixed model constant (*μ* = 0.5 in the simulations of Section 8); if online friction estimation was available, it could be updated without structural changes to either bound.

## 8. Experimental Validation

### 8.1. Experimental Setup and Pre-Calibration

**Figure 6:**
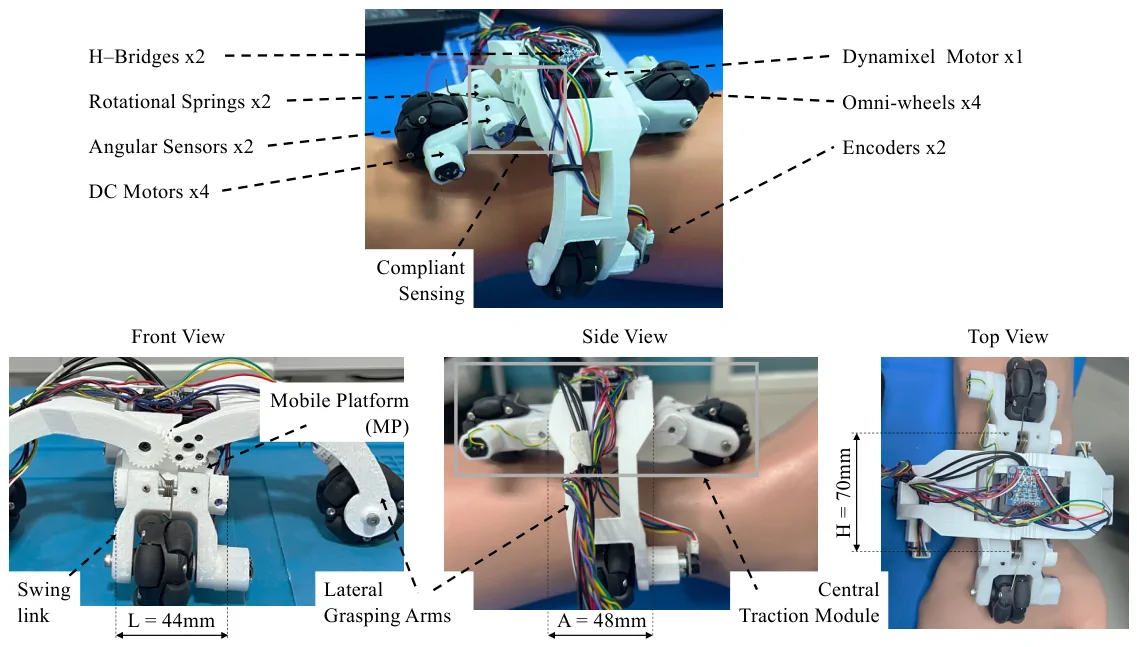
Isometric view showing the actuation and sensing systems. Front, side, and top views of the robot on a mannequin limb, highlighting its functional modules. The robot is shown mounted on a mannequin arm; no human subjects were involved in any of the tests.

The robot prototype has a mobile platform (MP) made from 3D-printed PETG plastic (see Figure 6). The platform, grasping mechanisms, and links were modelled using SolidWorks 2024 Student Edition; the resulting design parameters are listed in Table 1. The control parameters are given in-line where each is introduced: the friction coefficient *μ* = 0.5, a conservative value adopted for the model and the simulations (in the orbital tests of Section 8.4 the coefficient *assumed by the controller* is instead swept over $\hat{\mu }\in \left\{0.50,1.00,1.70,2.20\right\}$ to probe its effect on the commanded grasping torque), the orbital PI gains $K_{p}=8.0\;N\cdot m\cdot s/\mathrm{rad}$ and $K_{i}=0.5\;N\cdot m/\mathrm{rad}$, the differential torque saturation $\Delta \tau_{\max}=50\;\mathrm{mN}\cdot m$, and the central force band $\left(f_{n_{0},\min},f_{n_{0},\max}\right)\approx \left(1.0,6.0\right)\;N$ with target $f_{n_{0}}^{d}=3.0\;N$. The remaining parameters used in Section 6 and Section 7 but not yet given a numerical value are as follows. The grasping-torque floor and ceiling of Equation (44) are $\tau_{a,\min}=100\;\mathrm{mN}\cdot m$ and $\tau_{a,\max}=1.5\;N\cdot m$. The central force PI gains of Equation (39) are $K_{p}^{\left(0\right)}=20\;\mathrm{mN}\cdot m/N$ and $K_{i}^{\left(0\right)}=8\;\mathrm{mN}\cdot m/\left(N\cdot s\right)$. The lumped viscous damping coefficient of Equation (31) is $b_{v}=2\times 10^{-4}\;N\cdot m\cdot s/\mathrm{rad}$; the rolling-resistance term of the same equation is implemented with a single shared coefficient $c_{r}=0.01$ (dimensionless) applied to the two lateral contacts only, the central contact’s rolling resistance being neglected as sub-dominant. Both $I_{zz}$ and $d_{G}$ depend on the mass distribution and the grasp configuration rather than being fixed constants: the simulation of Section 8.3 places the centre of mass on the vehicle axis at the cylinder surface, $d_{G}=R=33.0\;\mathrm{mm}$, giving $I_{zz}=m\;d_{G}^{2}=3.05\times 10^{-4}\;\mathrm{kg}\cdot m^{2}$. The rationale for keeping $d_{G}$ small is discussed in Section 9.

Before the validation experiments, two pre-calibration procedures were carried out to identify the controller parameters that cannot be derived from the geometric model alone.

**Figure 7:**
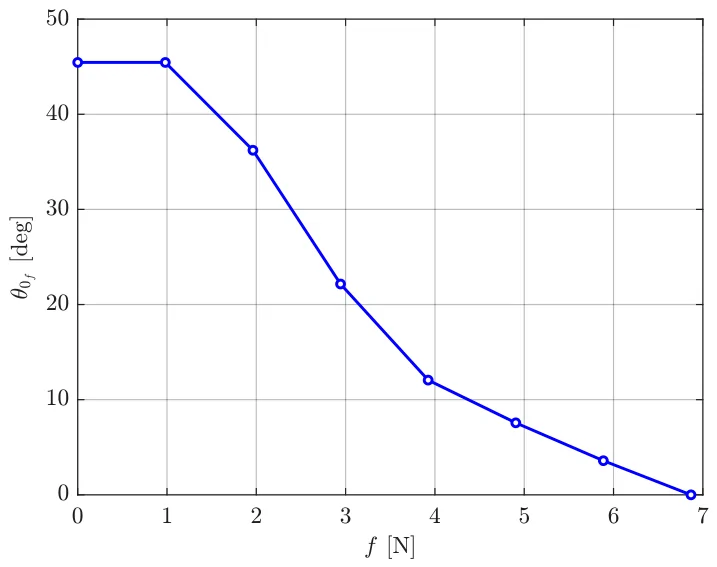
Experimental relationship between the applied gravitational force *f* and the angular deflection $\theta_{0}$ of one rotational spring of the swing link.

*Spring characteristic and contact monitoring.* Known masses were applied to one of the central wheels to load a single rotational spring of the compliant joint, and the corresponding angular deflection was measured with one angular sensor. For each applied load, *n* repeated measurements were recorded after the system reached steady state, and $\theta_{0}$ denotes the arithmetic mean of these measurements. The applied masses were converted into gravitational forces as $f_{n_{0}}=mg$, with $g=9.81\;m/s^{2}$. Figure 7 shows the resulting $f_{n_{0}}$–$\theta_{0}$ curve. Based on this calibration, the operational angular bound was set to $\theta_{0,\max}=40^{\circ }$, corresponding to approximately 6.25 N *per spring*; beyond this deflection the sensor voltage changes only slightly, indicating that the rotational spring has reached its physical compression limit. Since the transverse model lumps the two springs into one central contact (Section 4), the combined central force saturates at roughly twice this figure, about 12.5 N. The controller does not operate up to that limit: the ceiling of the admissible band of Section 7.2 is set conservatively at $f_{n_{0},\max}=6.0\;N$, keeping the estimate in the well-resolved part of the characteristic and the contact pressure modest, while the floor $f_{n_{0},\min}\approx 1.0\;N$ is the combined preload below which the deflection no longer resolves the load. Both bounds therefore derive from this calibration rather than from a tissue-safety criterion: they have not been checked against biomechanical pressure thresholds for skin or soft tissue, which would additionally require a contact-area model, and the tests reported here use a rigid cylinder. The calibration recorded steady-state readings after loading, without a load–unload cycle, so any hysteresis in the spring–sensor characteristic is not quantified; since the force estimate closes the PI loop, hysteresis would appear as a bias whose sign depends on loading history.

Both compliant joints and their angular sensors are nominally identical. The calibration characteristic G and the force estimate ${\hat{f}}_{n_{0}}$ refer to the force associated with a single rotational spring. Consistently with the lumped transverse model of Section 4, in which the central traction module reduces to the single contact $P_{c_{0}}$, the two readings are combined into one equivalent deflection *before* inversion: there is a single contact depth $a(\theta_{0})$ and therefore a single central force. After correcting the individual zero offsets, the mean zero-corrected deflection

(45) $${\bar{\Delta \theta }}_{0}=\frac{1}{2}\left(\Delta \theta_{0,f}+\Delta \theta_{0,b}\right)$$

is mapped onto the central normal force of the transverse plane,

(46) $${\hat{f}}_{n_{0}}=G^{-1}\;\left({\bar{\Delta \theta }}_{0}\right),$$

where $G^{-1}\left(\cdot \right)$ is the inverse of the measured characteristic, implemented through piecewise-linear interpolation. The front–rear difference is a longitudinal (pitch) effect and does not enter the transverse model. The bench loaded a single spring, so G and ${\hat{f}}_{n_{0}}$ above are per-spring quantities; the factor of two relating them to the lumped central force is applied when forming the band bounds, as noted above. These bounds $f_{n_{0},\min}$ and $f_{n_{0},\max}$ are the ones used by the radial force feedforward law (38) and, when the compliant sensor is available, by the PI correction (39). Even used only as a monitor, $\theta_{0}$ provides a coarse indication of contact status without requiring explicit knowledge of the spring stiffness.

### 8.2. Online Diameter Estimation: Sensitivity Analysis

The radius estimator (3) is a closed-form geometric function of the two measured angles $\theta_{a}$ and $\theta_{0}$; it requires no integration, filtering, or additional sensor. Its accuracy is therefore determined entirely by the partial sensitivities $\partial R_{\mathrm{est}}/\partial \theta_{a}$ and $\partial R_{\mathrm{est}}/\partial \theta_{0,i}$, i∈{f,b}, together with the resolution of the corresponding sensors, not by a simulation with arbitrary noise levels. We report a sensitivity study over the full physically admissible range.

#### 8.2.1. Physical Limits

Two geometric constraints bound the operating range; both are evaluated at the unloaded swing-link configuration $\theta_{0,f}=\theta_{0,b}=0$, for which $a=l_{4}+r_{w}=33\;\mathrm{mm}$. The minimum cylinder diameter corresponds to the configuration in which the two lateral wheels collide: $P_{1x}=r_{w}$, giving $\theta_{a,\max}=\arccos\;\left(\frac{r_{w}-l_{0}}{l_{1}}\right)=84.4^{\circ }$ and $D_{\min}=2R_{\min}=45\;\mathrm{mm}$. The maximum diameter is reached when the lateral contact climbs to the cylinder equator, $\gamma =90^{\circ }$, i.e., $P_{1x}=R+r_{w}$. Substituting (3) into this condition and simplifying yields a perfect square, ${\left(P_{1x}+P_{1y}+a-r_{w}\right)}^{2}=0$, so the equator is reached *tangentially*: *γ* attains $90^{\circ }$ without crossing it. The condition reduces to $l_{1}\left(\cos\theta_{a}-\sin\theta_{a}\right)=r_{w}-a-l_{0}$, giving the closed form

(47) $$\theta_{a,\min}=\arccos\;\left(\frac{r_{w}-a-l_{0}}{\sqrt{2}\;l_{1}}\right)-45^{\circ }=55.5^{\circ },$$

and $D_{\max}=2R_{\max}=87\;\mathrm{mm}$. Beyond this limit the contacts climb onto the upper hemisphere and the grip can no longer enclose the cylinder. The valid range is therefore $\theta_{a}\in \left[55.5^{\circ },\;84.4^{\circ }\right]$, corresponding to cylinder diameters D∈[45,87]mm, which spans the range of typical adult upper-limb segments.

#### 8.2.2. Sensitivity and Sensor-Limited Error

Differentiating (3) gives the partial sensitivities. Writing $u=P_{1y}+a\left(\theta_{0}\right)$, each swing-link term follows in closed form as

(48) $$\frac{\partial R_{\mathrm{est}}}{\partial \theta_{0,i}}=\frac{P_{1x}^{2}-{\left(u-r_{w}\right)}^{2}}{2\;{\left(u-r_{w}\right)}^{2}}\;\frac{l_{3}}{2}\cos\theta_{0,i},\;i\in \left\{f,b\right\},$$

which shows that each sensor contributes half of the offset gain, and the arm term $\partial R_{\mathrm{est}}/\partial \theta_{a}$ is obtained analogously. The total, first-order radius error is

(49) $$\delta R=\left|\frac{\partial R_{\mathrm{est}}}{\partial \theta_{a}}\right|\delta \theta_{a}+\sum_{i\in \{f,b\}}\left|\frac{\partial R_{\mathrm{est}}}{\partial \theta_{0,i}}\right|\delta \theta_{0,i}.$$

**Figure 8:**
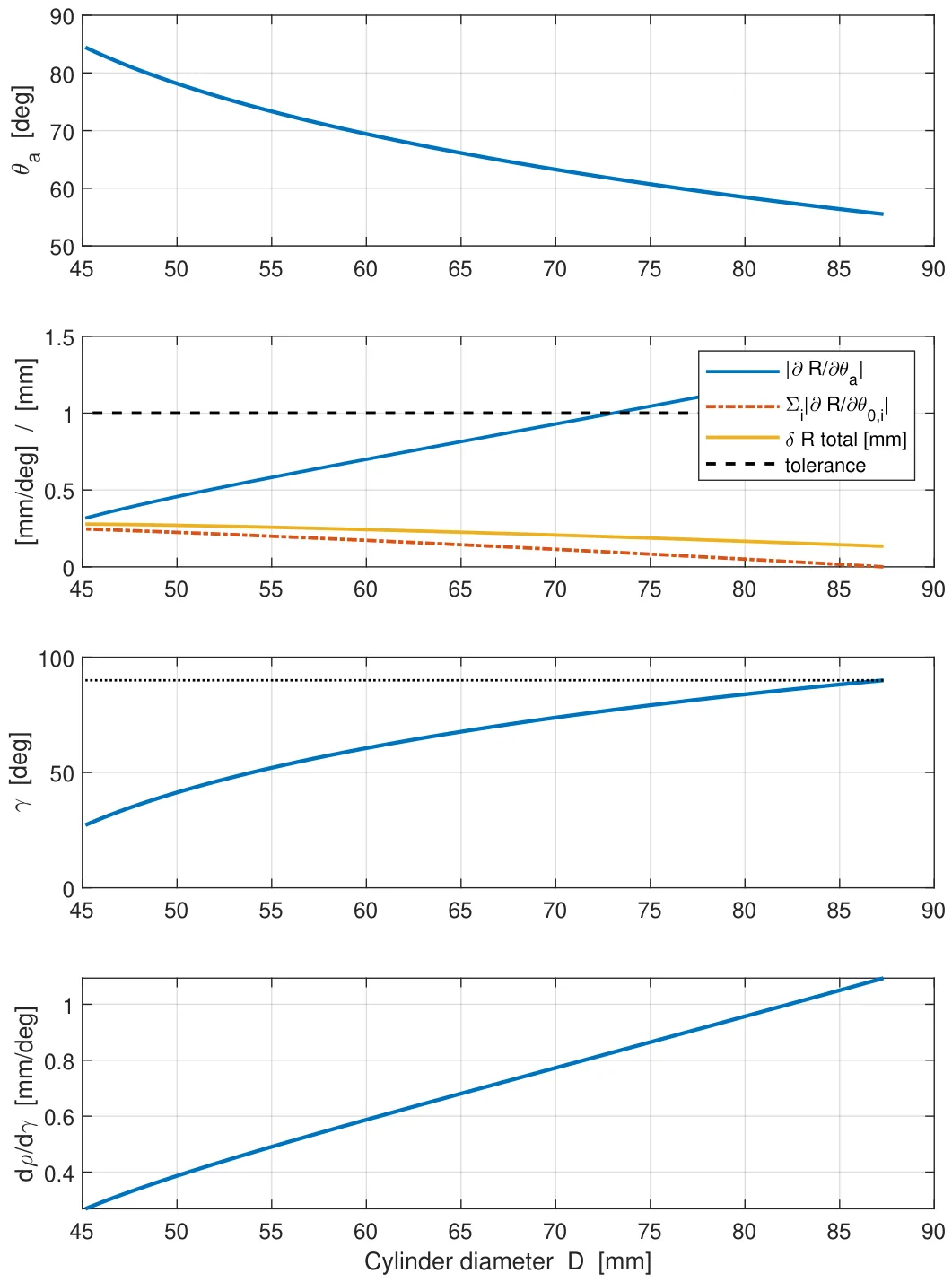
Sensitivity analysis of the online radius estimator (3) over the valid diameter range D∈[45,87]mm. From top to bottom: (i) required arm angle $\theta_{a}$ vs. cylinder diameter; (ii) estimator sensitivities $|\partial R_{\mathrm{est}}/\partial \theta_{a}|$ and $\sum_{i}\left|\partial R_{\mathrm{est}}/\partial \theta_{0,i}\right|$, together with the combined sensor-limited error (49), which stays below the 1 mm tolerance (dashed) throughout; (iii) lateral contact angle *γ*, which remains strictly within $\left(0^{\circ },90^{\circ }\right)$, confirming the grasp stays clear of both degenerate limits; (iv) radial force lever dρ/dγ of (9), which is strictly positive over the whole range, so the differential channel retains authority over the central contact force for every admissible cylinder.

The arm term is bounded by $\left|\partial R_{\mathrm{est}}/\partial \theta_{a}\right|\leq 1.31\;\mathrm{mm}/\deg$, so with the encoder resolution $\delta \theta_{a}=0.1^{\circ }$ it contributes at most 0.13 mm. The swing-link terms are bounded by $\sum_{i}\left|\partial R_{\mathrm{est}}/\partial \theta_{0,i}\right|\leq 0.25\;\mathrm{mm}/\deg$ (worst case at $\theta_{a,\max}$, i.e., on the smallest cylinder, and with both sensors erring in the same direction), so even coarse sensors with $\delta \theta_{0,i}=1^{\circ }$ add less than 0.25 mm. The total first-order error therefore peaks at 0.28 mm, well inside the 1 mm tolerance (relative error ≤ 0.6%) across the entire valid range. Note that the averaging in (1) is also what makes this bound conservative: independent errors on the two sensors partially cancel. Making $P_{c_{0}}$ a measured rather than a fixed quantity therefore costs little accuracy while providing the force feedback exploited in Section 7.2, and the estimator remains well-conditioned for all reachable cylinders. The sensitivities and the resulting error are shown in Figure 8. The lateral contact angle *γ* defined in (4) spans $27^{\circ }$–$90^{\circ }$ within the valid range, confirming that the contacts stay well clear of both degenerate limits for all admissible configurations, as required for orbital locomotion.

### 8.3. Closed-Loop Orbital Simulation

The complete control architecture of Section 7 is validated in simulation by integrating the orbital equation of motion (29) over a full revolution ($\phi :0^{\circ }\to 360^{\circ }$) using a trapezoidal velocity profile with 10% ramp-up, 80% cruise, and 10% ramp-down phases over $t_{\mathrm{end}}=20$ s. The orbital velocity controller is the PI loop with gravity feedforward defined in (35), whose output $\tau_{\Sigma }$ is split equally into $\tau_{w_{1}}^{\mathrm{ff}}=\tau_{w_{2}}^{\mathrm{ff}}=\tau_{\Sigma }/2$ and then combined with the differential radial force command $\Delta \tau_{d}$ through the allocation (34). The grasping torque feedforward is (44), clamped to $\left[\tau_{a,\min},\;\tau_{a,\max}\right]$.

**Figure 9:**
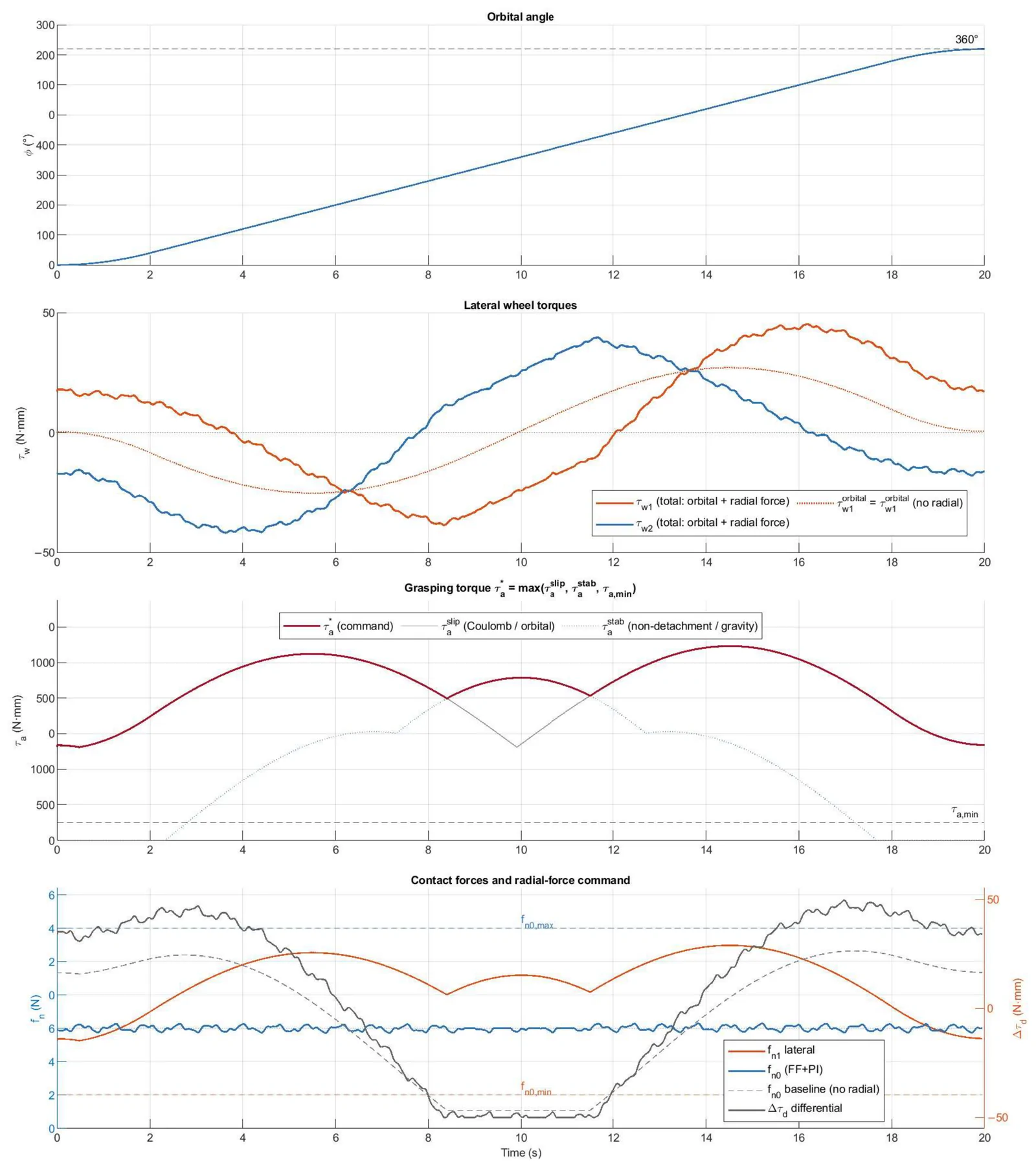
Closed-loop orbital simulation over one full revolution ($\phi :0^{\circ }\to 360^{\circ }$, $t_{\mathrm{end}}=20$ s, m=0.28 kg, μ=0.5, $d_{G}=R$), in the nominal configuration of the prototype, i.e., with the compliant sensor available and the PI correction active (${⊮}_{\mathrm{sensor}}=1$); the feedforward-only configuration is reported numerically in the table above but not plotted separately. From top to bottom: (1) orbital angle ϕ(t) following the trapezoidal velocity profile; (2) total lateral wheel torques $\tau_{w_{1}}$, $\tau_{w_{2}}$ (solid) against their orbital-only components with the differential offset $\pm \frac{1}{2}\Delta \tau_{d}$ removed (dotted); (3) commanded grasping torque $\tau_{a}^{*}$ (thick) as the upper envelope of the slip-prevention bound $\tau_{a}^{\mathrm{slip}}$ and the stability bound $\tau_{a}^{\mathrm{stab}}$ (thin), with minimum $\tau_{a,\min}$; (4) lateral contact force $f_{n_{1}}$ and central contact force $f_{n_{0}}$ with its baseline $f_{n_{0},0}$ and admissible band (left axis), and the sign-changing differential command $\Delta \tau_{d}$ (right axis).

The simulated plant is deliberately not identical to the model the controller inverts, so that the closed loop is not evaluated against its own assumptions. Two mismatches are injected. First, the radial coupling gain used by the feedforward law (38) is set 30% higher than the value that governs the simulated response, i.e., the controller inverts a model with $\hat{k}=1.3\;k$ where $k=\sin\gamma /r_{w}$; this represents uncertainty in *γ*, in the friction conditions and in the contact geometry, and it leaves a residual force error that pure feedforward cannot remove. Second, the force measurement fed to the PI correction (39) is corrupted by an additive non-harmonic disturbance of 0.15 N amplitude, representing sensor noise and unmodelled compliance in the swing links; this disturbance, and not a numerical artefact, is the source of the ripple visible in the lower panel of Figure 9. Running the same manoeuvre with the PI correction disabled (${⊮}_{\mathrm{sensor}}=0$) and enabled therefore quantifies what the correction contributes against a genuine, non-zero error (Table 2).

The feedforward term alone keeps the central force inside the admissible band over the whole revolution, so contact persistence does not depend on the force sensor; what the PI correction adds is accuracy about the set point. It removes the systematic offset left by the gain mismatch—a mean error of 0.157 N reduced to −0.008 N—and narrows the excursion by a factor of about four in both standard deviation and peak, holding $f_{n_{0}}$ within 0.15 N of its target throughout. This supports the architecture’s intended degradation property: the loop remains functional without the compliant sensor, at the cost of set-point accuracy rather than of contact maintenance. Both configurations are reproducible from the simulation script in the repository given in the Data Availability Statement.

Two limitations of this simulation should be stated. It is quasi-static in the radial direction: the differential torque is assumed to be reacted instantaneously by static friction at the lateral contacts, as discussed in Section 4.5, so the transient associated with the compliance of the arms and of the contact patch—and hence the bandwidth of the radial force loop—is outside the scope of the model. The PI gains of (39) are accordingly tuned to reject the steady-state offset rather than designed against an identified transfer function, and a formal stability margin for that loop would require identifying the stiffness and inertia of the arm–contact subsystem experimentally. This identification, and the closed-loop hardware validation it would enable, are the subject of ongoing work.

*Friction-cone utilisation and actuator limits.* Because (43) sizes the preload exactly at the Coulomb limit, the ratio $\max_{i}\left|f_{t,i}\right|/\left(\mu f_{n_{1}}\right)$ measures how much of the available friction the commanded torques consume, and hence how close the grasp runs to slip. With the mass distribution assumed here ($d_{G}=R$), the peak grasping torque demanded over the revolution is approximately 1.07N·m, i.e., some 29% below the actuator ceiling $\tau_{a,\max}=1.5\;N\cdot m$, so the bound is realisable throughout the orbit and the no-slip condition is met at the conservative value *μ* = 0.5 adopted for the model. The margin, however, is governed by the position of the centre of mass: since $\tau_{g}$ and hence the required preload scale linearly with $d_{G}$, the demanded torque reaches the actuator ceiling at $d_{G}\approx 59\;\mathrm{mm}$ (≈1.8R for this grasp), beyond which the command saturates and no-slip can no longer be guaranteed at *μ* = 0.5. A second, independent limit is set by the differential channel’s authority, $\left(\sin\gamma /r_{w}\right)\Delta \tau_{\max}=2.46\;N$, which bounds how far the baseline (37) may depart from the set point before Δτ saturates and $f_{n_{0}}$ can no longer be held at its target. With $d_{G}=R$ the baseline stays within that authority throughout the orbit, and the regulation reported above is achieved without saturating the channel; a larger $d_{G}$, or a grasp with smaller sinγ, would erode this margin as well as the actuator margin above.

Figure 9 shows the resulting time histories. Four observations are highlighted.

*Trajectory tracking.* The orbital angle ϕ(t) follows the commanded trapezoidal velocity profile, reaching $360^{\circ }$ at $t_{\mathrm{end}}=20$ s, with a visible acceleration phase in the first 2 s and a symmetric deceleration in the last 2 s.

*Common-mode and differential channels.* The total lateral wheel torques $\tau_{w_{1}}$ and $\tau_{w_{2}}$ (solid) are shown against their orbital-only components (dashed), obtained by removing the differential radial force offset $\pm \frac{1}{2}\Delta \tau_{d}$ (opposite sign on the two wheels). Both wheels change sign over the revolution, as expected for a common-mode gravity feedforward that reverses between the ascending and descending halves of the orbit. The separation between solid and dashed curves is the differential action, directly comparable to $\Delta \tau_{d}$ in panel 4; it is largest where the central contact most needs correction.

*Grasping torque.* The commanded $\tau_{a}^{*}\left(t\right)$ is the upper envelope of the two orientation-dependent bounds and the constant floor $\tau_{a,\min}$ of (44); the two bounds are plotted faintly beneath it: the slip-prevention term $\tau_{a}^{\mathrm{slip}}$ (Coulomb, driven by the orbital torque) and the stability term $\tau_{a}^{\mathrm{stab}}$ (lateral non-lift-off and central non-detachment, driven by gravity). The slip term forms the two outer lobes, peaking where the orbital torque is largest; the stability term produces the clean central bump around $\phi =180^{\circ }$ (mid-revolution), where the robot is inverted and gravity pulls the central contact force down hardest, so $\tau_{a}$ must rise to keep $f_{n_{0}}$ within reach of the differential channel. Between these regimes, $\tau_{a}^{*}$ falls to the floor $\tau_{a,\min}$ wherever the differential action alone suffices: the grasping actuator is engaged beyond its preload only when a stability bound requires it, yet never releases.

*Contact force regulation.* The lateral normal force $f_{n_{1}}$ follows $\tau_{a}^{*}$ proportionally through (16). The central contact force $f_{n_{0}}$ (blue) stays within the admissible band throughout the revolution, held close to its target $f_{n_{0}}^{d}$, whereas the uncontrolled baseline $f_{n_{0},0}$ (dashed grey) swings widely with orientation and would otherwise leave the band. The differential command $\Delta \tau_{d}$ (right axis) *changes sign* over the revolution: it relieves the central force (self-opening) where gravity already presses the central wheel against the surface, and increases it (self-closing) half a turn later. Because $\Delta \tau_{d}$ enters only the differential channel, this regulation leaves the orbital motion of panel 1 undisturbed, confirming the non-interference property of the actuation-space decomposition.

### 8.4. Preliminary Physical Prototype Tests

The following two tests were carried out on the physical prototype as a first, qualitative validation of the mechanism and the grasping-stability behaviour. They are *preliminary*: the platform is a single hand-built unit, the robot was moved manually around the fixed cylinder rather than by a commanded orbital trajectory, and no ground-truth force sensor was available at the central contact. They are therefore reported as proof-of-principle evidence that the modelled effects appear on hardware, not as a quantitative closed-loop validation, which remains future work.

The scope of these tests is best stated explicitly. They demonstrate the enclosure geometry and passive grasp retention across a wide span of orbital orientations, the ability of the mechanism to orbit the cylinder under wheel actuation, and the qualitative dependence of the commanded grasping torque on orientation and on the assumed friction coefficient, including the detachment failure mode when that coefficient is over-estimated. Four claims are left untested. The differential channel is not exercised independently, since the lateral wheels are driven in open loop with equal, same-direction torques, so the orthogonality of the two actuation channels is not measured; the PI correction of (39) is not engaged, as no ground-truth force sensor was available at the central contact; and the radius estimator of Section 4.3 is not compared against externally measured diameters. These four claims rest on the model and on the simulation of Section 8.3 alone. Validating them requires a closed-loop test bench with an instrumented cylinder providing independent force and diameter references, which is the subject of ongoing work.

**Figure 10:**
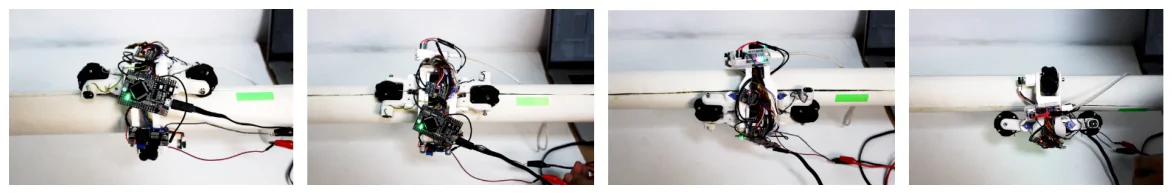
Sequence of screenshots (from left to right) showing the robot orbiting around the fixed cylinder while retaining its grasp, at $0^{\circ }$, $45^{\circ }$, $90^{\circ }$ and $180^{\circ }$. The green square serves as a fixed visual reference for the robot’s orbital rotation.

*Grasp retention under orbital rotation.* The prototype weighs 280 g including all electronics (H-bridges, sensors and motors). It was closed around a D=56mm cylinder by the grasping servomotor (DYNAMIXEL XC330-T288-T, ROBOTIS Inc., Seoul, South Korea) under a fixed current set point, and the robot was then moved manually around the fixed cylinder from approximately $0^{\circ }$ to $\approx -135^{\circ }$. The robot retained the grasp and remained seated on the surface throughout this range of orientations (Figure 10), confirming that the enclosure geometry of Section 4 holds over a wide span of orbital angles. Contact was monitored through the swing-link sensors, the orbital angle being estimated from the IMU accelerometer components as $\phi =\mathrm{atan}2(A_{X},A_{Z})$.

**Figure 11:**
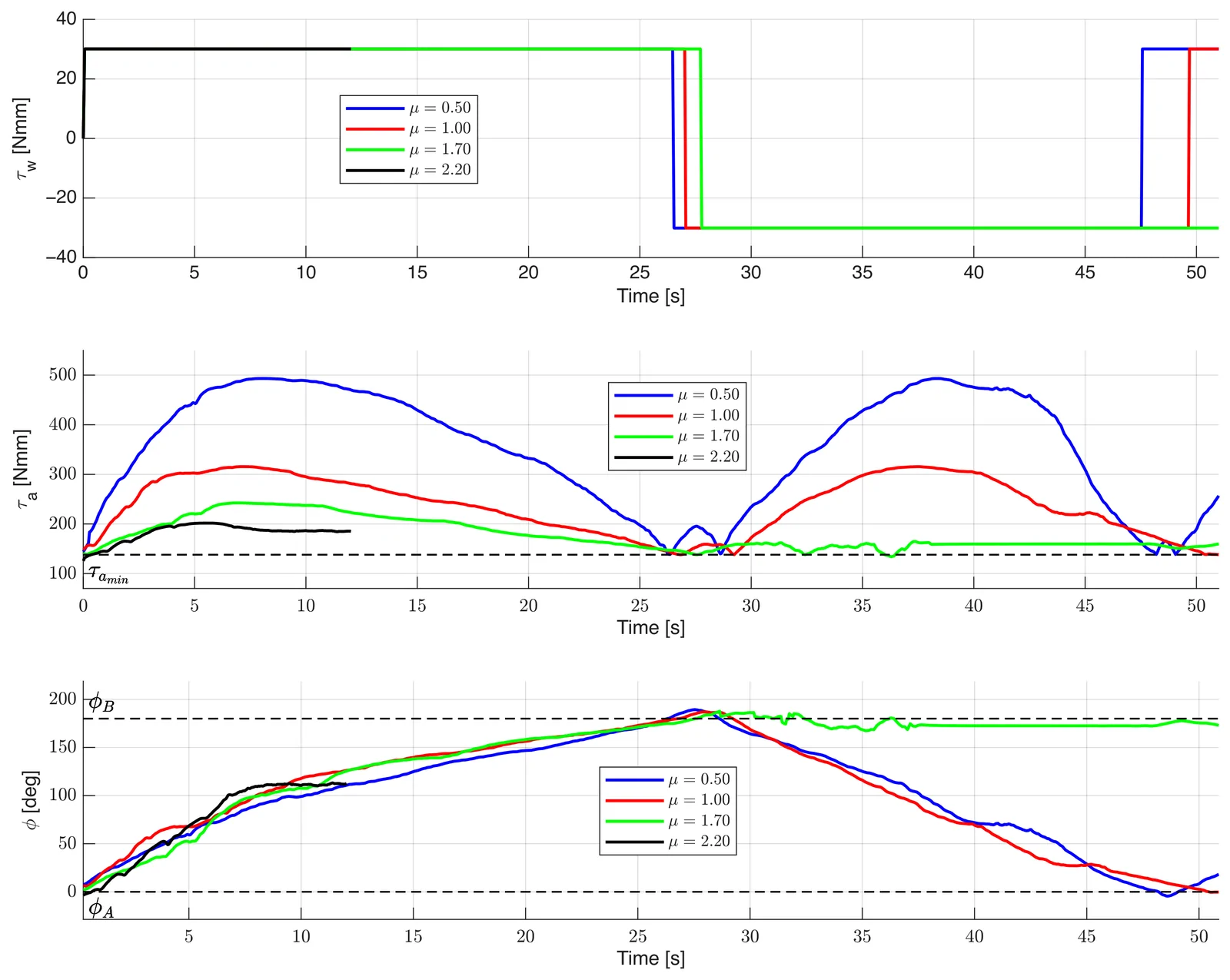
Time evolution of the main actuation and contact variables during the open-loop orbital rotation test, for four values of the friction coefficient $\hat{\mu }$ *assumed by the controller* when evaluating the grasping–torque bounds (the physical surface is the same across runs). From top to bottom: the lateral wheel torque $\tau_{w}$ (equal same-direction command, its sign reversed at $\phi_{B}$ for the return), the grasping torque $\tau_{a}$ computed online from *ϕ* via (41)–(43), and the orbital angle *ϕ* estimated from the IMU. For the black curve ($\hat{\mu }=2.20$) the controller over-estimates friction and therefore commands too small a $\tau_{a}$, so the grasp is lost and the vehicle detaches and falls before completing the orbit.

*Orbital rotation.* Orbital rotation motion tests of the robot around a fixed cylinder were conducted, as shown in Figure 11. The lateral wheels were driven in *open loop* with equal, same-direction (common-mode) torques, so that the platform orbits from its initial configuration ($\phi_{A}=0^{\circ }$) toward the prescribed target ($\phi_{B}=180^{\circ }$). Once the orbital angle estimated by the IMU reaches $\phi_{B}$, the sign of the lateral wheel torque $\tau_{w}$ is reversed (top subplot) to command the return manoeuvre back to $\phi_{A}$. The orbital angle is obtained directly from the accelerometer as $\phi =\mathrm{atan}2(A_{X},A_{Z})$, and the grasping torque $\tau_{a}$ is computed online from *ϕ* through the stability and slip bounds (41)–(43).

The four runs differ only in the friction coefficient $\hat{\mu }$ *assumed by the controller* when evaluating (41)–(43); the true surface friction is identical in all of them. Because those bounds make $\tau_{a}\propto \left|\sin\phi \right|/\hat{\mu }$, a larger assumed $\hat{\mu }$ commands a smaller grasping torque, and hence a smaller normal force margin. For the lower assumed values the commanded $\tau_{a}$ keeps the grasp within its safe band and the platform completes the forward-and-return manoeuvre (bottom subplot). The black curve ($\hat{\mu }=2.20$) shows the failure mode of friction over-estimation: the assumed coefficient is far larger than the real one, so the commanded $\tau_{a}$ is too low to sustain the required normal force, the central contact detaches, and the vehicle slips and falls under gravity before reaching $\phi_{B}$.

The middle subplot shows the grasping torque $\tau_{a}$, which the controller modulates with orientation to compensate the tangential component of the robot’s weight, following the $\tau_{a}\propto \left|\sin\phi \right|/\hat{\mu }$ relationship implied by (41)–(43). The same law is visible in the over-estimated run: after the grasp is lost, as the detached robot swings further around the cylinder, the |sinϕ| factor changes accordingly and the commanded torque follows it, confirming the real-time execution of the gravity compensation law.

## 9. Conclusions

This paper presented an open, non-anthropomorphic on-limb robot that orbits a cylindrical surface while actively regulating its grip. The central result is an actuation-space decomposition in which the two lateral wheel torques, written in a common-mode/differential basis, simultaneously drive the orbital motion and regulate the central normal force without mutual interference: the same basis diagonalises both the velocity Jacobian and the static force map, so the differential channel is provably orthogonal to orbital propulsion. This lets a single pair of actuators do the work that would otherwise require a dedicated radial force mechanism.

Regulation is performed by a model-based feedforward law, obtained by inverting the static contact model, corrected by a PI term fed back from the spring-loaded swing links of the central traction module. In a full-revolution closed-loop simulation the differential command *reverses sign* over the orbit—relieving the central force where gravity already presses the wheel against the surface and increasing it half a turn later—keeping $f_{n_{0}}$ inside its admissible band while the grasping torque returns to its minimum wherever the differential action alone suffices, and without perturbing the orbital trajectory.

The same compliant mechanism plays a double role: it makes the central contact point a measured rather than a fixed quantity—at a cost of less than 0.25 mm in radius-estimation accuracy—and, in exchange, supplies the force measurement that closes the radial force loop with no dedicated force transducer. From only two proprioceptive signals, the arm encoder and the swing-link deflections, a closed-form estimator recovers the cylinder radius and the three contact points with sub-millimetre accuracy across the full 45–87 mm diameter range. The orthogonality result and the estimator accuracy are established analytically and, for the closed loop, in simulation; the hardware evidence is qualitative. Preliminary tests on the physical prototype support the model qualitatively: the grasp is retained across a wide span of orbital orientations, and the commanded grasping torque follows the predicted $\tau_{a}\propto \left|\sin\phi \right|/\hat{\mu }$ law; over-estimating the friction coefficient assumed by the controller under-commands the grasp and, beyond a critical value, loses traction altogether.

Taken together, these mechanisms give the robot a rudimentary form of the active haptic perception humans use when they grip and slide a hand along another person’s limb: it senses the limb’s size through the grasp itself and continuously regulates how hard it holds on, in the same safety-margin regime observed in arboreal gripping. This makes the platform a concrete, if simplified, engineering instance of enclosure-based haptic exploration coupled to locomotion, and a step toward on-body robots that move along a limb as unobtrusively as a guiding hand.

One design implication follows from the model rather than from the particular prototype. The gravitational torque about the cylinder axis, $\tau_{g}=-m\;g\;d_{G}\sin\phi$, scales linearly with the distance $d_{G}$ from that axis to the vehicle’s centre of mass, and so does, through the slip-prevention bound (43), the grasping torque the actuator must supply over the whole orbit. Keeping the centre of mass close to the cylinder axis is therefore a primary design constraint: for the prototype reported here the demanded torque reaches the actuator ceiling at $d_{G}\approx 1.8\;R$, and a design that exceeds it cannot guarantee slip-free operation without a more powerful grasping actuator—which, being heavier and bulkier, would itself raise *m* and typically $d_{G}$, tightening the same bound it was meant to relax. A compact mass distribution wrapped closely around the limb is thus doubly favourable, and it is compatible with the same enclosure geometry that the grasp requires: the constraint $d_{G}\leq R$ that places the centre of mass within the support triangle (Section 5.3) also leaves a comfortable actuator margin. This consideration should guide the placement of batteries and electronics in future revisions, which in the present unit were positioned without regard to it.

Several directions present themselves for future work. The feedforward term alone already achieves stable regulation under nominal parameters, and the PI correction becomes decisive when parametric uncertainty—in the friction coefficient *μ*, the geometric coupling gain $\sin\gamma /r_{w}$, or unmodelled contact geometry—would otherwise push the true central force outside the admissible band. Characterising the resolution and hysteresis of the spring-based force estimate, and quantifying how much of the residual error it can actually remove across a wider range of operating conditions, is a natural extension of the analysis in Section 8.3. Beyond that, online estimation of the friction coefficient *μ* and full closed-loop orbital position control are natural next steps toward a clinically deployable system.

## Figures and Tables

**Table 1:** Mechanical design parameters of the experimental prototype.

Parameter	Symbol	Value
Wheel radius	$r_{w}$	19 mm
Lateral arm pivot x-offset	$l_{0}$	10 mm
Lateral arm length (pivot to wheel centre)	$l_{1}$	93 mm
Swing-link length	$l_{3}$	30 mm
Swing-link pivot y-offset	$l_{4}$	14 mm
Total vehicle mass	*m*	0.28 kg

**Table 2:** Central force regulation over one full revolution, with the PI correction disabled and enabled. Both configurations use the same injected model-gain mismatch and measurement disturbance.

	Feedforward Only	Feedforward + PI
$f_{n_{0}}$ range [N]	2.43–3.54	2.86–3.15
Mean tracking error [N]	0.157	−0.008
Standard deviation [N]	0.383	0.062
Peak deviation [N]	0.567	0.147
Samples within band	100%	100%

## Data Availability

The MATLAB/Octave simulation code, the CAD design files, and the numerical parameters used throughout this manuscript are openly available at https://github.com/TaISLab/Open-limb-robot (accessed on 31 July 2026).
